# Mechanisms of Luoshi Neiyi prescription (LSNYP) in endometriosis: a network pharmacology and experimental study

**DOI:** 10.1186/s41065-026-00637-2

**Published:** 2026-01-19

**Authors:** Lizheng Wu, Rui Su, Jinjin Jia, Zijun Kuang, Cheng Zeng, Fangli Pei

**Affiliations:** 1 The First Clinical Medical College, Guangzhou University of Chinese Medicine, Guangzhou, Guangdong 510405 China; 2 Department of Gynecology, Guangzhou Hospital of Integrated Traditional Chinese and Western Medicine, Guangzhou, Guangdong 510800 China; 3 Department of Traditional Chinese Medicine, Qinghai University Medical College, Xining, Qinghai 810016 China; 4 The First People’s Hospital of Foshan, Foshan, 528000 Guangdong China; 5 Department of Gynecology, the First Affiliated Hospital of Guangzhou University of Chinese Medicine, Guangzhou, Guangdong 510405 China

**Keywords:** Endometriosis, Adhesion-invasion-angiogenesis, Luoshi neiyi prescription, Traditional chinese medicine, Network pharmacology

## Abstract

**Background:**

Luoshi Neiyi prescription (LSNYP) is a traditional Chinese medicine that has a clinical effect on endometriosis (EMs). This study combined network pharmacology with experimental validation to explore its potential molecular mechanisms.

**Methods:**

The primary components of LSNYP were identified based on the Traditional Chinese Medicine Systems Pharmacology Database and Analysis Platform (TCMSP) and a Bioinformatics Analysis Tool for Molecular Mechanism of Traditional Chinese Medicine (BATMAN-TCM). The possible target proteins were predicted using the SwissTargetPrediction online tool. The GeneCards and DisGeNET databases were used to identify targets associated with EMs. The protein-protein interaction (PPI) network, herb-component-target network, Gene Ontology (GO) and Kyoto Encyclopedia of Genes and Genomes (KEGG) pathway enrichment analyses were performed. Molecular docking, molecular dynamics (MD) simulation and experimental verification were carried out.

**Results:**

217 potential therapeutic targets were identified. Enrichment analyses revealed involvement in key biological processes and pathways, including cell migration, inflammatory response, focal adhesion, and the VEGF signaling pathway, which are closely related to the adhesion-invasion-angiogenesis progression in EMs pathogenesis. Molecular docking and MD simulation results showed stable binding between corresponding components and typical targets (ICAM1, MMP9 and VEGFA) involved in the progression. Experimental results demonstrated that LSNYP could decrease typical targets of the progression in rats and inhibit the invasion, migration and adhesion capabilities of human endometriotic stromal cells (ESCs).

**Conclusion:**

These findings suggest LSNYP may be a promising candidate for EMs, potentially through inhibiting the adhesion-invasion-angiogenesis progression.

**Supplementary Information:**

The online version contains supplementary material available at 10.1186/s41065-026-00637-2.

## Introduction

Endometriosis (EMs) is a disease characterized by endometrial tissue growth and infiltration outside the uterine cavity [1]. The clinical manifestations of EMs include infertility, dysmenorrhea, chronic pelvic pain, dyspareunia, pelvic mass, and even malignant transformation [2]. Despite its benign nature, ectopic endometrial tissue exhibits tumor-like biological properties, including migration, adhesion, invasion, angiogenesis, and implantation [3]. Population-based studies have demonstrated that EMs is linked to an increased risk of clear cell ovarian carcinoma (CCOC); concurrently, molecular investigations have identified microRNA-874 as a tumor suppressor and potential therapeutic target in the progression from EMs to CCOC [4, 5]. However, EMs pathogenesis remains complex, with many theories including Sampson’s retrograde menstruation theory, coelomic metaplasia theory, hormonal theory, and immunological theories; the precise molecular mechanism requires further clarification. Currently, the main treatments for EMs include nonsteroidal anti-inflammatory drugs, combined hormone-related drugs and surgery, those are effective at ameliorating pelvic pain and lesion development. However, there are also some side effects, and the recurrence rate is high after the treatment is stopped [6]. Therefore, exploring the mechanisms of malignant behaviors and developing novel multi-targeted treatments for EMs are critical.

Complementary and alternative medicine is a potentially valuable source of new treatments. Traditional Chinese medicine (TCM) has proven to be safe and effective as an alternative therapy for EMs [7]. According to the theories of TCM, chi-promoting, blood-activating and stasis-removing approaches are the basic therapeutic strategies for EMs. Luoshi Neiyi prescription (LSNYP, also known as Yimu Tiaojinghuayu Mixture) consists of fourteen Chinese herbal medicines (Supplement File 1) and was invented based on above principles. Previous studies have demonstrated its clinical efficacy. For instance, Gao et al. found no significant difference in the overall trend of postoperative carbohydrate antigen 125, visual analog scale (*P* > 0.05) and recurrence rate (*P* = 0.530) between the LSNYP group and oral contraceptive group [8]. Liu found that LSNYP combined with ultrasonic interventional therapy could reduce the recurrent ovarian endometriotic cyst size [9]. Prior experimental studies have shown that LSNYP can inhibit the inflammation-estradiol (E_2_) cycle, improve hypoxia, and enhance endometrial receptivity of eutopic endometrium in rat models [10–12]. Nevertheless, the mechanisms underlying the action of LSNYP remain incompletely characterized due to its complexity. Whether LSNYP exerts therapeutic effects through additional pathways, such as suppression of malignant biological behaviors, remains to be elucidated.

**Fig. 1:**
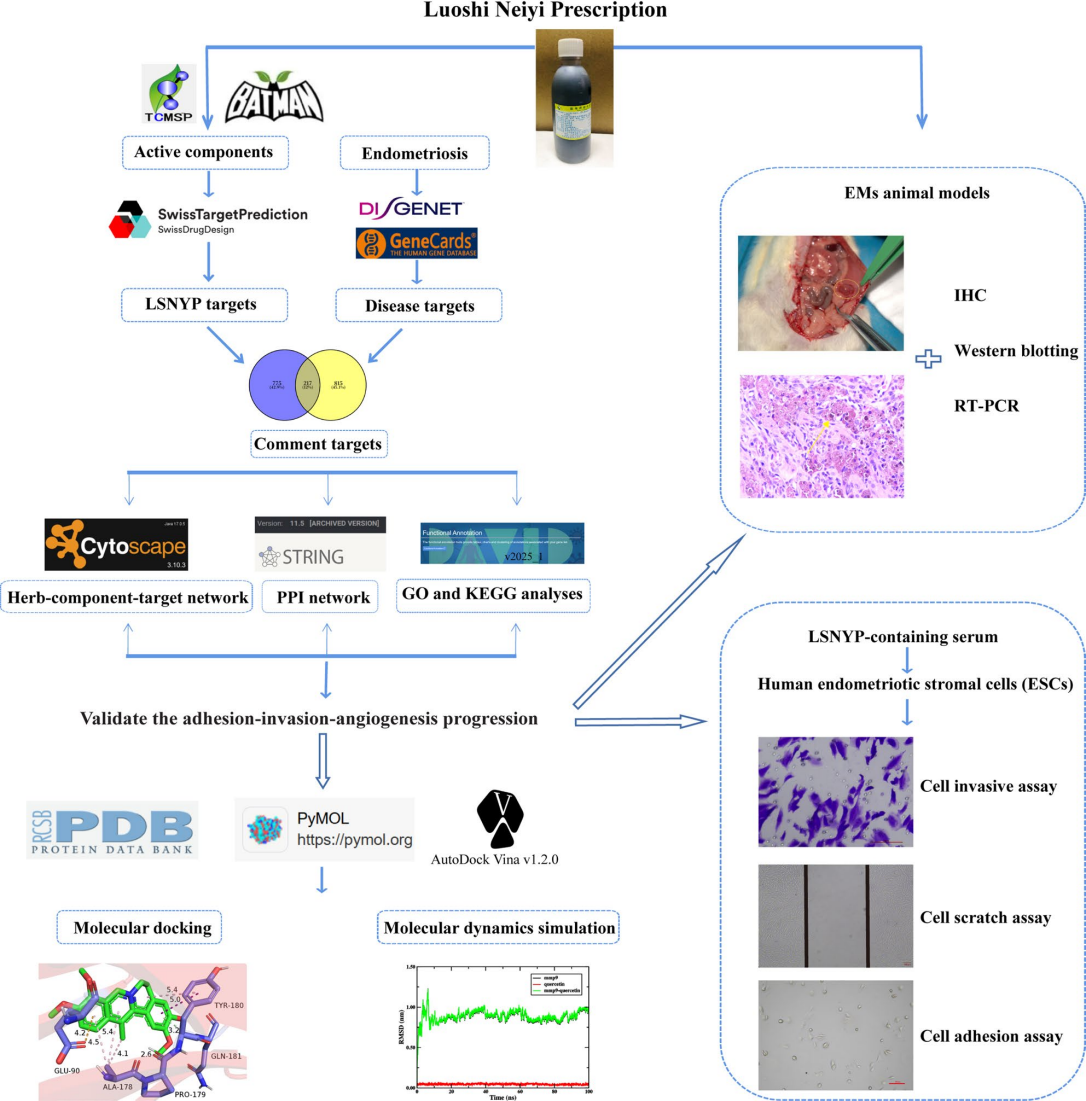
The Workflow of the study

It is well established that a fundamental characteristic of TCM is its synergistic effect, achieved through the combination of various botanical, mineral, or animal-derived components, to effectively treat complex diseases and syndromes. Network pharmacology can integrate molecular biology, pharmacology and computer science [13] and explain interactions among molecular regulatory networks. This approach is widely employed to comprehensively analyze TCM formulas [14, 15] and provides a unique advantage in elucidating the mechanisms [16, 17]. Therefore, we employed network pharmacology to construct an herb-active component-target network and performed molecular docking and experiments to verify certain targets implicated in the adhesion-invasion-angiogenesis cascade in EMs. These results are anticipated to provide novel mechanistic insights into LSNYP’s therapeutic action against malignant biological behaviors in EMs. A flowchart is shown (Fig. 1).

## Materials and methods

### Identification of the active components of LSNYP

We used the Traditional Chinese Medicine Systems Pharmacology Database and Analysis Platform (TCMSP, https://old.tcmsp-e.com/tcmsp.php; accessed on May 10, 2022) [18] and a Bioinformatics Analysis Tool for Molecular Mechanism of Traditional Chinese Medicine (BATMAN-TCM, http://bionet.ncpsb.org.cn/batman-tcm/; accessed on May 10, 2022) [19] to collect information on the chemical components of LSNYP. In the TCMSP, molecules with oral bioavailability (OB) ≥ 30% and drug-likeness (DL) value ≥ 0.18 were selected [20]. Similarly, according to the parameter settings recommended by the database and the prevalent literature, the following screening criteria were applied: *p* value < 0.05 and score > 20 in BATMAN-TCM [21, 22]. Therefore, the active compounds of YMC, CX, TR, DS, PH, YHS, WY, HZ, ZBM and WM were identified in the TCMSP, and those in TBC, SZ, WLZ and ML were found in BATMAN-TCM. Later, components with a canonical simplified molecular-input line-entry system (SMILES) structure in the PubChem database were exported.

### Identification of component and disease-associated targets

The canonical SMILES structures of the active components were successively imported into the SwissTargetPrediction online tool (http://www.swisstargetprediction.ch/; accessed on January 12, 2022) [23]. After the species was set to “human”, targets with a probability greater than 10% were identified. The keyword “endometriosis” was input into GeneCards Database (https://www.genecards.org/; accessed on January 14, 2022) and the DisGeNET database (https://www.disgenet.org/; accessed on January 14, 2022) [24, 25]. Due to the high number of disease targets, we set targets with relevance scores ≥ 0.84 (top 50%) in the Gene Cards database as primary targets in EMs. Moreover, the inclusion criterion was a Gene-Disease Association (GDA) score ≥ 0.3 (medium-high confidence) based on the DisGeNET database, and the EMs-associated targets were identified after duplicates were deleted. Finally, the Venny 2.1 online tool (https://bioinfogp.cnb.csic.es/tools/venny/index.html) [26] was used to generate a diagram of the intersection of component-associated targets and EMs-associated targets, and the overlapping target genes were considered the potential therapeutic targets of LSNYP in EMs.

### Construction of the herb-component-target network and protein-protein interaction (PPI) network

The potential therapeutic targets were imported into STRING database (version 11.5, https://cn.string-db.org/, accessed on September 3, 2025) [27, 28], with the species set as “Homo sapiens” and the minimum interaction confidence threshold set to “medium confidence (0.400)”. Then the network was visualized and analyzed using Cytoscape 3.10.3 software [29]. Topological parameters, including degree, betweenness centrality, and closeness centrality, were calculated using the built-in NetworkAnalyzer tool.

### Gene ontology (GO) and Kyoto encyclopedia of genes and genomes (KEGG) enrichment analyses

Potential therapeutic targets of LSNYP in EMs were analyzed using the DAVID bioinformatics database (https://david.ncifcrf.gov/summary.jsp; accessed on September 4, 2025) [30]. Using “Homo sapiens” as the background species, GO and KEGG pathway enrichment analyses were performed. The top ten significantly enriched terms or pathways, ranked by false discovery rate (FDR), were selected and visualized using the Wei sheng xin online platform (http://www.bioinformatics.com.cn).

### Molecular docking

We used the RCSB Protein Data Bank database (https://www.rcsb.org/) to obtain the crystal structures of target proteins [31], the screening conditions [32] were as follows: (1) the species source was limited to “Homo sapiens”; (2) the resolution is high (resolution ≤ 2.5 Å); (3) the protein structure scanned by the X-ray single crystal diffraction; (4) structure (single-stranded, double-stranded or other) depended on the number of polymers of the protein in the organism. PyMOL (version 2.3.0) software was used to remove water molecules, original ligands and unnecessary protein chains [33]. Meanwhile, the structures of components were obtained from the PubChem database [34], and they were imported into Chem3D (version 2020) software for energy minimization and optimization [35]. After that, AutoDockTools (version 1.5.6) software [36] was utilized to add hydrogen atoms to optimized proteins and ligands, and torsion bonds of the small molecule ligands were also selected. The active site (a binding pocket) of protein was predicted by using POCASA 1.1 online tool [37] to prepare for molecular docking. AutoDock Vina (version 1.2.0) software was then used for docking [38], the Lamarckian genetic algorithm and semi-flexible docking were chosen, the default exhaustiveness was set to 8, and the maximum output was set to 9. The docking protocol generated 9 binding poses per run, and the structure with the lowest binding energy was selected for docking with the target protein, and results were visualized.

### Molecular dynamics (MD) simulation

MD simulation of the protein-ligand complex was performed with GROMACS 2025.2 software package. The system was placed in a periodic cubic box, solvated with transferable intermolecular potential with 3 points (TIP3P) water and described by the Chemistry at HARvard Macromolecular Mechanics (CHARMM) force field version 36; ions were added to achieve a physiological ionic strength (150 mM NaCl). After energy minimization, the system was equilibrated sequentially in the canonical ensemble and isothermal-isobaric ensemble (100 ps each) with position restraints. A 100-ns production run was then carried out with a 2-fs time step. After removal of periodic-boundary artifacts and recentering the system, trajectories were analyzed for root-mean-square deviation (RMSD) and radius of gyration (Rg) of the protein backbone, per-residue root-mean-square fluctuation (RMSF), solvent-accessible surface area (SASA), and the number of protein-ligand hydrogen bonds.

### Animal treatments and Preparing LSNYP-containing serum

Thirty 2-month-old female rats weighing 200 ± 20 g were obtained from the Experimental Animal Center of Guangzhou University of Chinese Medicine. All animals were housed under standard controlled conditions with free access to standard rodent diet and water. After 1 week of acclimatization, the rats were intramuscularly injected with E_2_ benzoate (0.05 mg/kg/day, 210301, Shanghai quanyu Biotechnology, China) for 5 days to synchronize their estrous cycles. Using a random number table, all rats were first divided into two groups based on body weight: one group consisting of 20 rats and another of 10 rats. The group of 20 rats underwent autotransplantation to establish the EMs model, following a previously described procedure by Gokaip et al. [39, 40]. Postoperative penicillin (QM2201201, North China Pharmaceutical Co., Ltd, China) and E_2_ benzoate treatments continued for 5 days. The remaining 10 rats were assigned to the sham-operated group and only underwent laparotomy. Four weeks post-modeling, the 20 modeled rats were again randomly assigned to either the model group or the LSNYP group (10 rats per group).

Each bottle of LSNYP (batch number: Yue Yao zhi Z20071247, Guangzhou Kangyuan Pharmaceutical Co., Ltd.) contained 250 mL, and for adult female patients, 75 mL/d per 60 kg of body weight is orally administered. A 200 g rat corresponds to 0.018 times that of a 70 kg adult, as per the body surface area conversion factors detailed by Nair and Jacob [41], so the clinical equivalent dose for a rat was calculated to be 0.78 mL/100 g. Considering the relatively low dosage of each herbal component in the prescription and accounting for metabolic differences between species, as well as double the dose was recommended as the starting dose in the previous literature [42, 43], so the LSNYP treatment group was administered a doubled volume of 1.56 mL/100 g/day, this solution was subsequently concentrated to a final volume of 0.78 mL/100 g to ensure feasible intragastric delivery. Rats in the sham and model groups received intragastric administration of saline at the same volume of 0.78 mL/100 g/day. After 4 weeks of continuous feeding, all the rats were intraperitoneally anesthetized with avertin and euthanized, and blood was collected from the abdominal aorta, lesions were measured using the ellipsoid formula (V = 1/2 × length × width²) [44]. The EcM and eutopic endometrium (EuM) were then removed (the normal endometrium was harvested from rats in the sham group) for immunohistochemistry, western blotting and semi-quantitative reverse transcription-polymerase chain reaction (RT-PCR).

Ten additional rats were randomly allocated to control and LSNYP groups (5 rats per group). The LSNYP group received the double dose of LSNYP via oral gavage, twice daily for five days, while the control group was given normal saline. One hour after the last administration, all rats were anesthetized and blood was collected from the abdominal aorta. Serum was obtained after clotting and centrifugation (3000 rpm, 10 min), inactivated at 56 °C for 30 min, filtered through a 0.22 μm membrane, and stored at − 80 °C for subsequent cell experiments.

### Enzyme-linked immunosorbent assay (ELISA)

Rat serum was centrifuged at 3000 rpm for 10 min at 4 °C, and the supernatant was collected. Alanine aminotransferase (ALT, 05850797190, Cobas) and creatinine (CREA, CH0101066, Maccura) were measured on a Roche Cobas c801 automated biochemical analyzer with automatic concentration calculation [45].

### Immunohistochemistry

The endometrial tissues were fixed with 10% formalin solution and sectioned. After antigen retrieval in citrate buffer, sections were incubated with 10% normal goat serum (AR1009, Boster) for 30 min to block nonspecific antibody binding, with primary antibodies against ICAM1 (1:100, MA5407, Thermo fisher), MMP9 (1:1000, ab76003, Abcam), or VEGFA (1:500, ab39250, Abcam) at 37°C for 1 h and with secondary antibodies at 37°C for 30 min. The sections underwent a washing procedure with phosphate-buffered saline (PBS) for 2 min per wash, repeated five times. Subsequently, sections were developed using 3,3’-diaminobenzidine (DAB, G1212, Servicebio) for 2 min, counterstained with hematoxylin for 1 min, differentiated in hydrochloric acid-alcohol for 5 s, and then blued under running tap water for 2 min. After mounting with a suitable medium, the stained tissue samples were visualized with a PANNORAMIC slide scanner, and Alpathwell v2 (Servicebio, CHN) was used to calculate the histochemistry score (H-score) as ∑ (pi × i) = (percentage of weak intensity area × 100) + (percentage of moderate intensity area × 200) + (percentage of strong intensity area × 300) [46]. In this formula, pi represents the percentage of pixels with a positive signal, and i represents the grade of the positive signal. The H-score ranged from 0 to 300, and larger values indicated a stronger comprehensive staining intensity [47, 48].

### RT-PCR analysis

Total RNA was isolated from tissues using the total RNA extraction kit (LS1040, Promega), followed by reverse transcription into complementary DNA (cDNA) using the Revertaid First Strand cDNA Synthesis Kit (K1622, Thermo Fisher). PCR amplification was performed on a T-1 Thermoblock (Biometra) using Taq DNA Polymerase (B500010-0200, Sangon Biotech). The protocol was as follows: an initial pre-denaturation at 97 °C for 3 min, followed by 30 cycles of denaturation at 97 °C for 20 s, annealing at 60 °C for 15 s, and extension at 72 °C for 24 s, and a final extension at 72 °C for 3 min. The reaction was held at 12 °C until analysis. The primers used are listed in Table 1. The final relative mRNA level was calculated by separating 20 µL of the PCR product by 1.5% agarose gel electrophoresis and observing the bands with an ultraviolet transilluminator [49].

**Table 1:** Primer sequence for rat

Gene	Primer sequence (5’→3’)
*ICAM1*	F: CAGGTGGGCAAGAACCTCAT
	R: TGTCGAGCTTCAGGACCCTA
*MMP9*	F: GCATCTGTATGGTCGTGGCT
	R: CGTGCGGGCAATAAGAAAGG
*VEGFA*	F: GGGAGCAGAAAGCCCATGAA
	R: TGCGCTTTCGTTTTTGACCC
*ACTB*	F: GCAGGAGTACGATGAGTCCG
	R: GGGTGTAAAACGCAGCTCAG

### Western blotting

Total protein was extracted by lysing tissue samples in radio-immunoprecipitation assay (RIPA) lysis buffer (P0013B, Beyotime) supplemented with 1% protease (P105539, Aladdin) and phosphatase (S1873, Beyotime) inhibitor cocktail. After the protein concentration was measured using the bicinchoninic acid (BCA, P0010, Beyotime) method, 40 µg of protein from each sample was separated by sodium dodecyl sulfate‒polyacrylamide gel electrophoresis (SDS–PAGE). The proteins were then transferred to polyvinylidene difluoride (PVDF, IPVH00010, Millipore) membranes at 200 mA for 90 min (for GAPDH and VEGFA) or 200 mA for 120 min followed by 300 mA for 15 min (for MMP9 and ICAM1), which were blocked with 5% skim milk for 2 h at room temperature on a shaker. The membranes were then incubated overnight at 4 °C with polyclonal antibodies against GAPDH (1:1000, AB-P-R 001, Goodhere), ICAM1 (1:500, 16174-1-AP, Proteintech), MMP9 (1:1000, AF5228, Affinity), and VEGFA (1:500, AP0742, Bioworld). After 5 washes, the membranes were incubated with corresponding secondary antibody (1:50000, BA1054, Boster) at 37 °C for 2 h, washed again 5 times. Immunoreactive bands were visualized using an enhanced chemiluminescence (ECL, P1050, Applygen) substrate solution, and the gray values of the bands in the photographs were analyzed using Image-Pro Plus (IPP) software [50].

### Cell culture

Primary human endometriotic stromal cells (ESCs) were isolated from ovarian endometriotic cysts of patients in accordance with the method of Guan in our department [51]. Briefly, tissues were minced and digested with type I collagenase (1 g/mL, C0130, Sigma) at 37 °C for 60 min. Digestion was terminated with complete medium, and the mixture was sequentially filtered through 100- and 400-mesh sieves. ESCs were collected from the filtrate via centrifugation at 1000 rpm for 10 min. The cells were resuspended and cultured at 37 °C with 5% CO₂, and passages 3–5 were used for experiments.

### Cell counting kit-8 (CCK-8) assay

Cells plated in 96-well plates were allocated into four groups: ESCs (10% blank serum), 2% LSNYP (10% medicated serum: blank serum = 1:4), 6% LSNYP (10% medicated serum: blank serum = 3:2), and 10% LSNYP (10% medicated serum: blank serum = 1:0). After serum treatment for 24, 48, and 72 h, 10 µL of CCK-8 reagent (K1018, APExBIO) was added to each well, and the optical density (OD) at 450 nm was measured. Cell viability was calculated as follows: (OD of medicated serum − OD of blank well) / (OD of ESCs − OD of blank well) × 100%.

### Cell invasion assay

Cells were treated with drug-containing serum for 72 h, harvested, and adjusted to 3 × 10⁵ cells/mL. The lower chamber of a 24-well plate was filled with 800 µL of complete medium. The upper chamber of Transwell (353097, Falcon) was coated with 100 µL Matrigel (0.5 mg/mL, 356234, Corning) and allowed for gel formation. Then, 200 µL of cell suspension was added to the upper chamber and incubated for 24 h at 37 °C with 5% CO₂. After incubation, non-invading cells were gently removed. The remaining invading cells were fixed with 70% ice ethanol for 1 h, stained with 0.5% crystal violet (G1014, Servicebio) at room temperature for 20 min, and then photographed using an inverted microscope (Ta2-FL, Nikon).

### Cell scratch assay

Following treatment with drug-containing serum for 72 h, cells were harvested and seeded into 6-well plates. Upon reaching over 90% confluence, a straight scratch was generated perpendicular to the pre-marked lines using a sterile pipette tip. After washing, serum-free medium was added. Images were captured immediately (0 h) and after 24 h of incubation.

### Cell adhesion assay

Cells were treated with drug-containing serum for 72 h, harvested, and adjusted to 6 × 10^4^ cells/mL. 50 µL Matrigel (1 mg/mL) was pipetted into the center of each well in a 96-well plate and polymerized at 37 °C for 4–5 h. 100 µL of cell suspension was added and incubated for 1 h at 37 °C with 5% CO₂. Finally, the unadhered cells were washed away and the remaining cells were photographed.

### Quantitative real-time polymerase chain reaction (qPCR)

After 72 h of treatment with drug-containing serum, cells were harvested for analysis. Total RNA was isolated from the cells utilizing the TRIzol reagent (15596-026, Ambion). Reverse transcription was carried out with the HiFiScript All-in-one RT Master Mix (CW3371M, Cwbio) under the following conditions: 50 °C for 15 min and 85 °C for 5 s. Primers specific for human genes (Table 2) were procured from Tsingke Biotech. qPCR was performed on an ABI ViiA™ 7 Real-Time PCR System employing the AceQ qPCR SYBR Green Master Mix (Q111, Vazyme), following this cycling protocol: an initial pre-denaturation at 95 °C for 10 min, succeeded by 40 cycles of denaturation at 95 °C for 10 s and a combined annealing/extension phase at 60 °C for 60 s. After amplification, a melting curve was generated using a three-step protocol: 95 °C for 15 s, 60 °C for 60 s, and 95 °C for 15 s. Finally, relative mRNA levels were determined using the 2^−ΔΔCt^ method.

**Table 2:** Primer sequence for human

Gene	Sequence (5’→3’)	Gene	Sequence (5’→3’)
*ICAM1*	F: CAGAGGTTGAACCCCACAGT	*EGFR*	F: GCGCTACCTTGTCATTCAGG
	R: TCTGAGACCTCTGGCTTCGT		R: TATCAATGCAAGCCACGGTG
*MMP9*	F: GCTACCACCTCGAACTTTGAC	*IL-1β*	F: CGAATCTCCGACCACCACTA
	R: TCAGTGAAGCGGTACATAGGG		R: AGCCTCGTTATCCCATGTGT
*VEGFA*	F: GGAGGAGGGCAGAATCATCA	*STAT3*	F: AGAAAACATGGCTGGCAAGG
	R: CTTGGTGAGGTTTGATCCGC		R: GCCTCCTTCTTTGCTGCTTT
*IL-6*	F: AGGAGACTTGCCTGGTGAAA	*SRC*	F: CTACTACTCCAAACACGCCGAT
	R: CAGGGGTGGTTATTGCATCT		R: TCCTCTGAAACCACAGCATACA
*TNF-α*	F: TCAGAGGGCCTGTACCTCAT	*MAPK3*	F: TCAACACCACCTGCGACCTTAA
	R: GGAAGACCCCTCCCAGATAG		R: GTACCAGCGCGTAGCCACATA
*Akt1*	F: ACACCAGGTATTTTGATGAGGAG	*GAPDH*	F: TCAAGAAGGTGGTGAAGCAGG
	R: TCAGGCCGTGCCGCTGGCCGAGTAG		R: TCAAAGGTGGAGGAGTGGGT
*TP53*	F: AGGTTGGCTCTGACTGTACC		
	R: GATTCTCTTCCTCTGTGCGC		

### Statistical analysis

The data analyses were performed using SPSS 27.0, and bar graphs were generated with GraphPad Prism 9.0. Normality was assessed with the Shapiro-Wilk test. Normally distributed data are expressed as mean ± standard deviation (SD), comparisons between two groups were conducted using independent sample *t*-test, and comparisons among multiple groups were performed using one-way analysis of variance (ANOVA). For multiple comparisons, if homogeneity of variance was assumed, the least significant difference (LSD) post hoc test was applied; otherwise, Tamhane’s T2 test was used. Non-normally distributed data are presented as median (interquartile range, P25–P75) and were analyzed using the Kruskal–Wallis test. *P* < 0.05 was considered to indicate statistical significance.

## Results

### Potential therapeutic targets of LSNYP in EMs

**Fig. 2:**
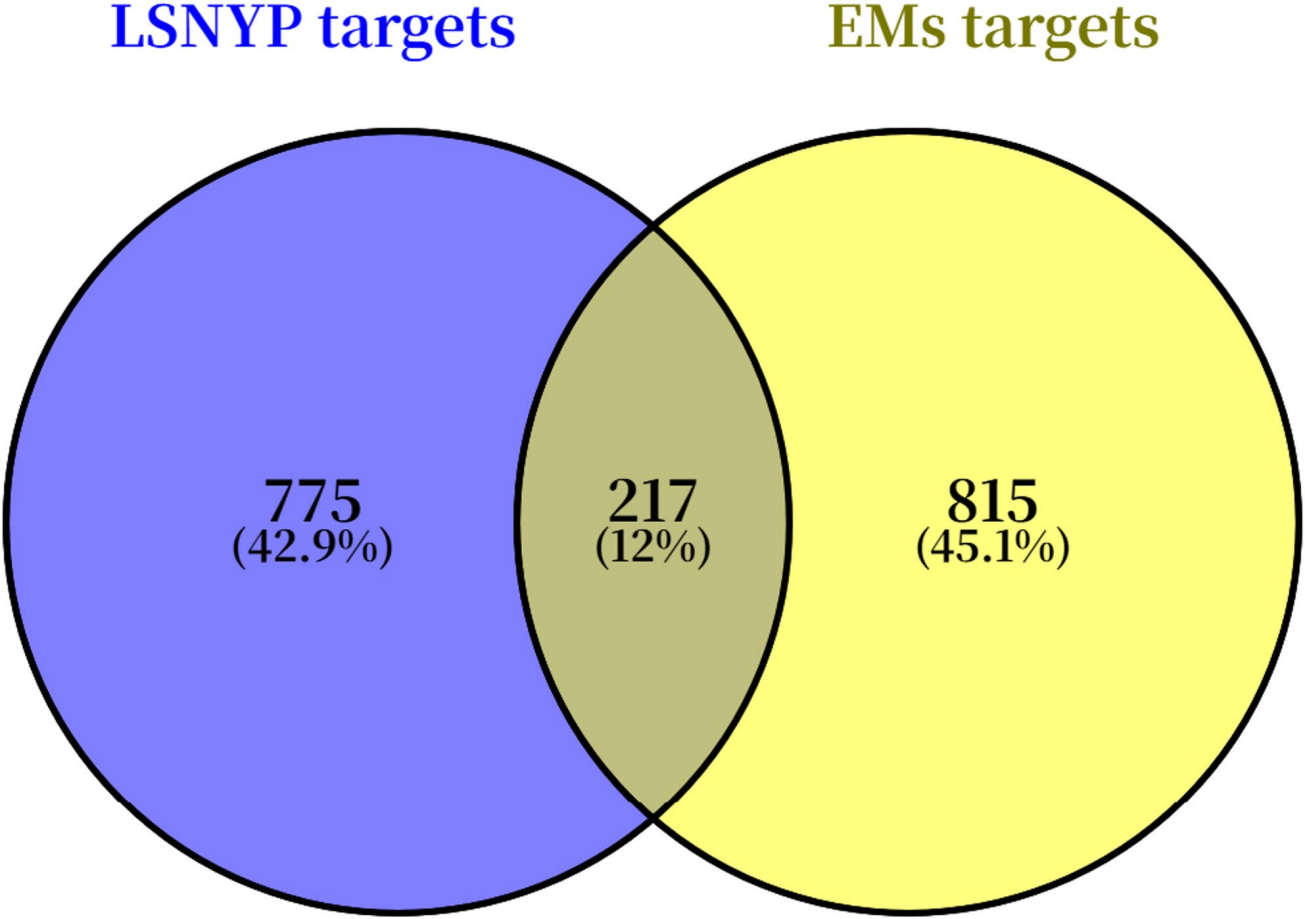
Venn diagram of LSNYP targets and EMs-related targets

After duplicates were removed, 114 components of LSNYP were identified from the TCMSP and BATMAN-TCM databases (Supplement File 2). A total of 992 proteins targeted by the 114 candidate active components were predicted by the SwissTargetPrediction online tool. In addition, 929 and 165 EMs-related targets were identified from the GeneCards and DisGeNET databases, respectively, and 1032 remained after merging the data and deleting duplicates. A Venn diagram (Fig. 2) was then drawn, and 217 potential therapeutic targets of LSNYP in EMs were identified (Supplement File 3).

### Herb-component-target network

**Fig. 3:**
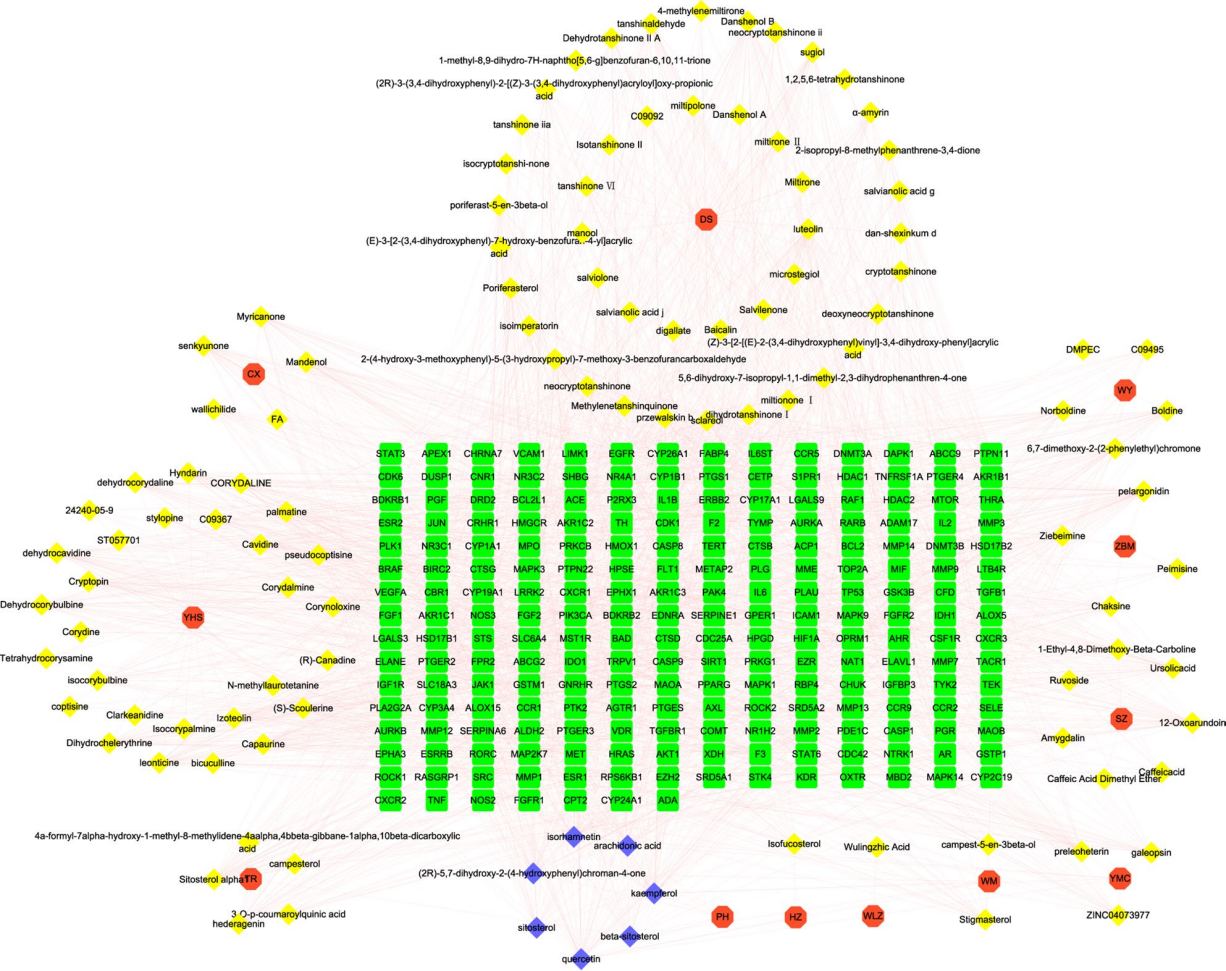
Herb-component-target network. Notes: The red hexagon represents herb, the yellow and blue rhombus represent component, the green square represents target

The herb-component-target network was visualized as shown in Fig. 3. We calculated the total degree of targets acted on by each component and computed the average value for all components. On average, each component was linked to 17 targets, with a higher degree value indicating a greater number of potential target associations. The top five components based on degree values in the intersection network were quercetin, isorhamnetin, kaempferol, luteolin, and pelargonidin, all of which exhibited degrees ≥ 43, suggesting their potential importance in the treatment of EMs. Similarly, the degree of components associated with each target was summarized and averaged. The results showed that 76 targets, including CYP19A1, MMP9, Akt1, PTGS2, ESR2, and EGFR, were found to have above-average corresponding components (mean = 9). These results demonstrate that LSNYP exerts its therapeutic effects through multiple components acting on multiple targets.

### PPI network of potential therapeutic targets

**Fig. 4:**
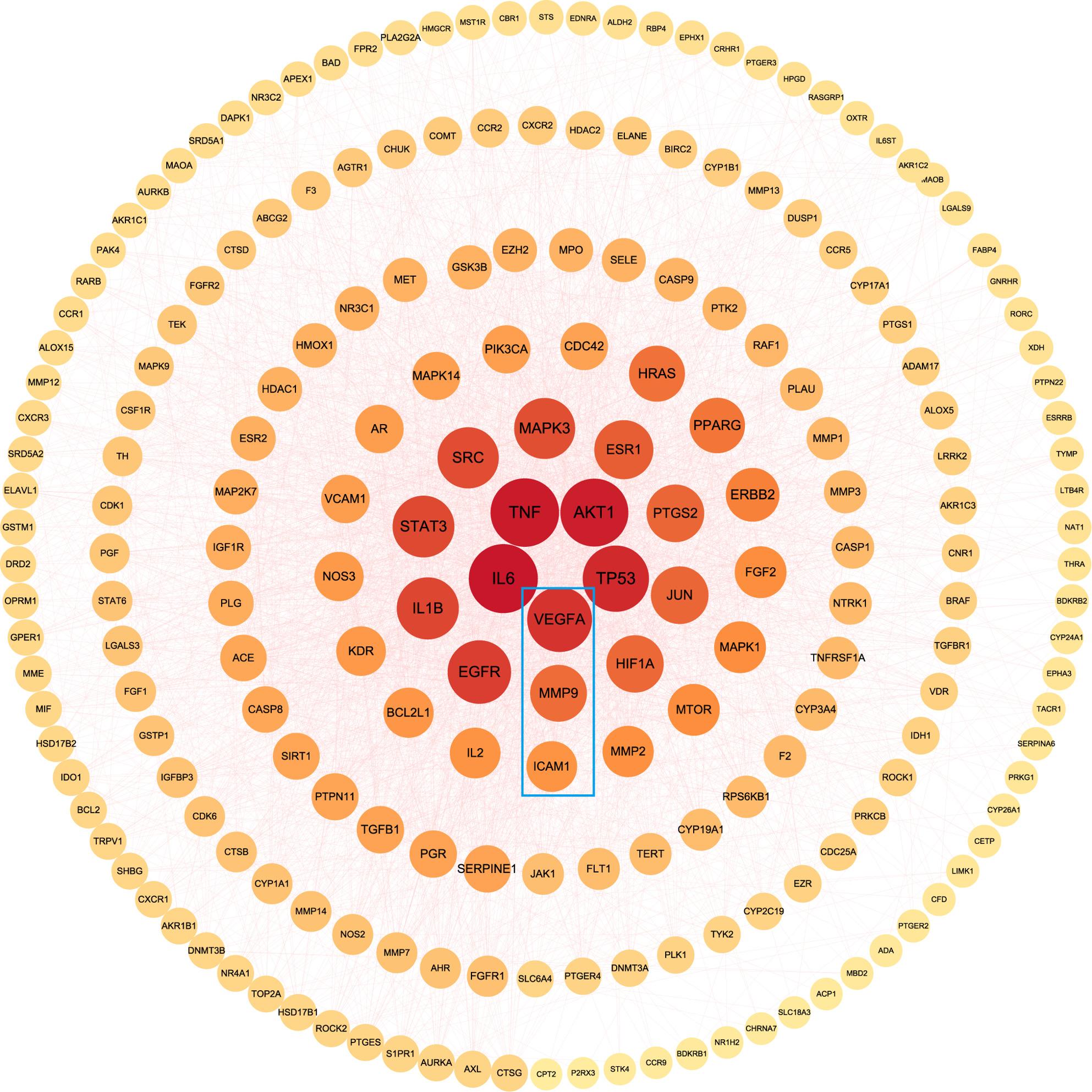
PPI network of key hub targets. Notes: The larger the node and the brighter the color are, the higher the degree value

A total of 217 potential therapeutic targets were entered into the STRING database, and the obtained information was visualized in Fig. 4, with nodes sorted by degree value. Core targets were further refined using selection criteria requiring a degree value greater than twice the median (46), and both betweenness centrality and closeness centrality above the median (0.001009, 0.5175). This process identified the top 46 in the PPI map as core targets, including IL-6, TNF, ICAM1, MMP9, and VEGFA, among others. These proteins are likely to serve as critical hubs within the network, bridging interconnected nodes and acting as central targets for the treatment of EMs with LSNYP.

### GO and KEGG enrichment analyses

**Fig. 5:**
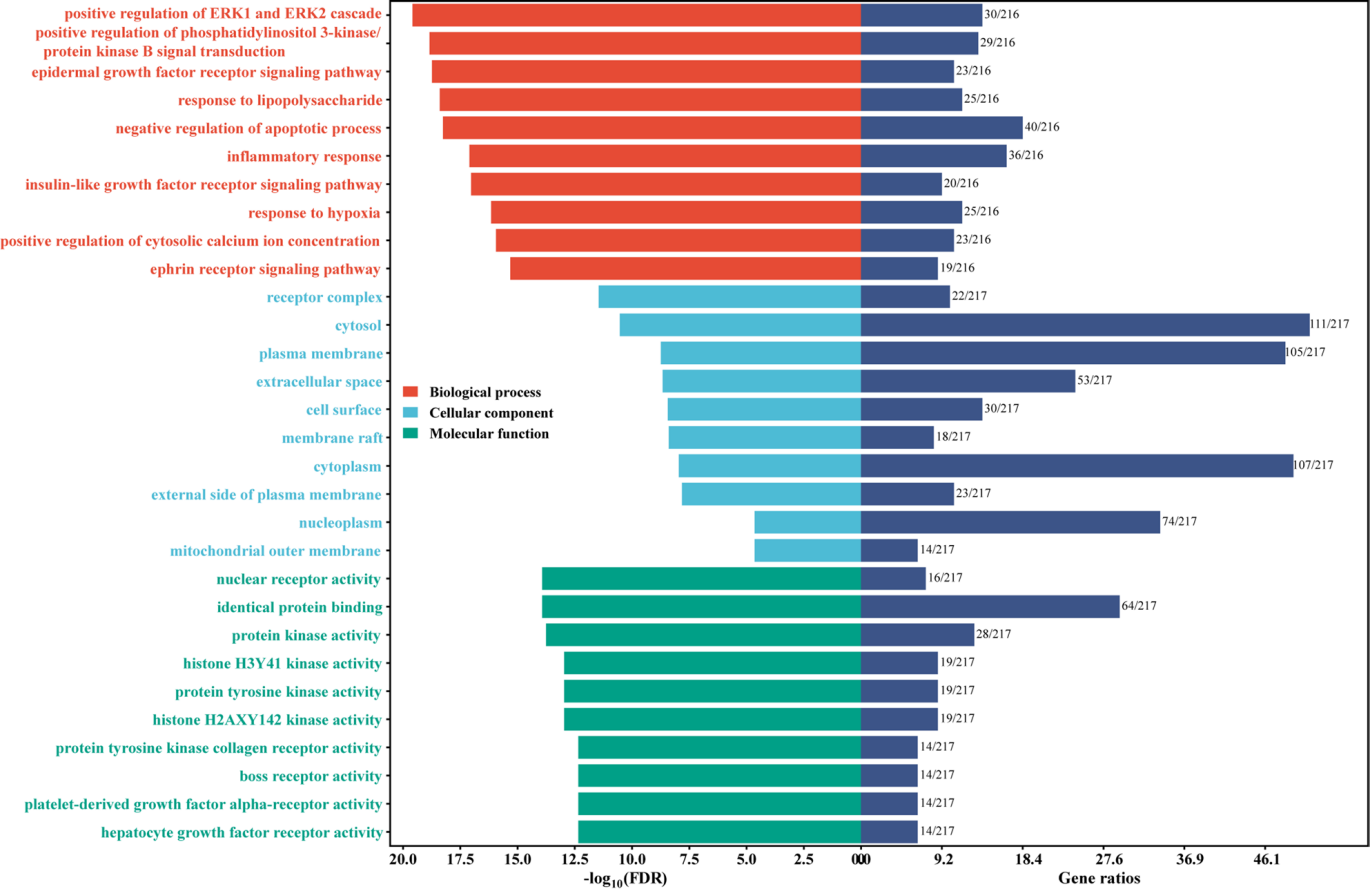
Bar chart of GO enrichment analysis (Top 10)

**Fig. 6:**
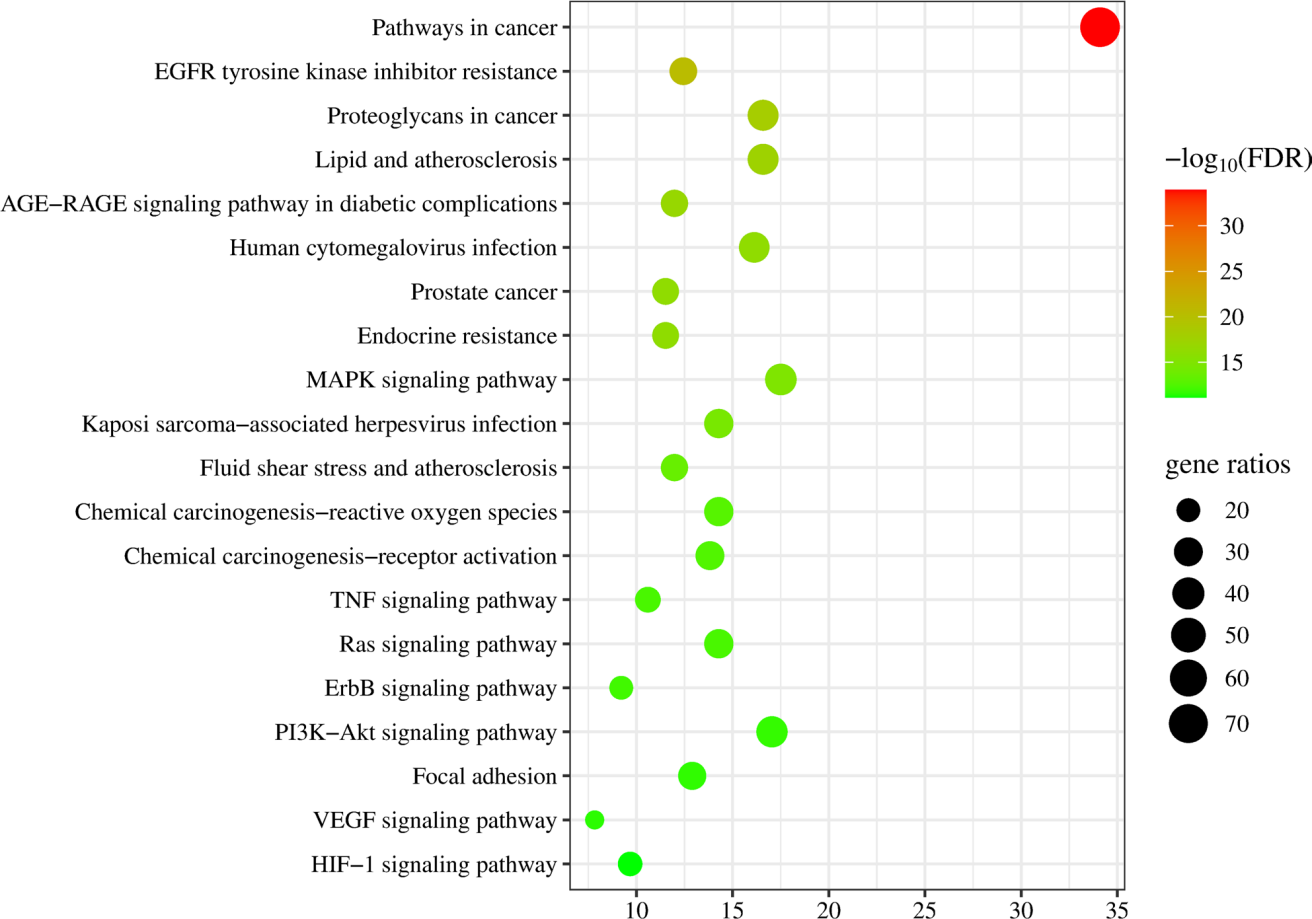
Bubble diagram of top KEGG pathways (Top 30). Note: Bubble size represents the Gene Ratio. Color intensity represents the statistical significance based on − log_10_ (FDR)

To further investigate the mechanism through which LSNYP affects EMs, the 217 targets were investigated using the DAVID bioinformatics database. After eliminating results with a low correlation, the top 10 significant biological process (BP), molecular function (MF), and cellular component (CC) terms and KEGG pathways were visualized based on the FDR value (Figs. 5 and 6). According to the GO enrichment analysis, the 453 BP terms included inflammatory response, response to hypoxia, and cell migration, etc.; the 44 CC terms included receptor complex, cell surface, and focal adhesion, etc.; and the 187 MF categories focused on nuclear receptor activity, identical protein binding, and vascular endothelial growth factor receptor activity, etc. The enriched KEGG pathways included endocrine resistance, focal adhesion, EGFR tyrosine kinase inhibitor resistance, and the VEGF, HIF1A, MAPK, and TNF signaling pathways, etc.

Recent studies suggest that the adhesion-invasion-angiogenesis progression plays an important role in the implantation and development of EMs [52]. Each step in the process requires the participation of a series of cytokines and enzymes, and among these, ICAM1, MMP9, and VEGFA are the most representative factors of the three stages, respectively [53]. Inhibition of the adhesion-invasion-angiogenesis progression is therefore key to the treatment of EMs [54]. In the network pharmacology study, ICAM1, MMP9, and VEGFA were identified as core targets, and they were usually also closely linked to these star pathways, such as NF-κB, TNF, PI3K-Akt pathways and so on. The functional analysis also demonstrated that LSNYP could regulate multiple related biological processes and pathways, such as inhibition of the proliferation, adhesion, and angiogenesis pathways. Based on these findings, we hypothesized that LSNYP alleviates EMs by inhibiting the adhesion-invasion-angiogenesis progression. Thus, the three key protein ICAM1, MMP9 and VEGFA were selected for further research and verification.

### Molecular docking visualization

All components corresponding to ICAM1, MMP9 and VEGFA in the herb-component-target network were selected for molecular docking with the corresponding proteins. The information of proteins is shown in Table 3. To validate the protocol, the co-crystallized ligands of ICAM1 (NAG) and VEGFA (SO4) were re-docked; MMP9 was excluded due to the absence of a native ligand. The resulting RMSD values were all 0 Å and below 2.0 Å, confirming the robustness of the docking protocol. Furthermore, metformin was employed as a negative control and yielded binding energies of − 4.6, − 5.8 and − 4.3 kcal/mol for ICAM1, MMP9 and VEGFA, respectively. These values were higher than 99% of test results (Table 4), confirming specific and favorable ligand binding. It is generally believed that a lower binding energy is associated with stronger binding activity between the receptor and ligand and greater stability [55]. The results showed all the binding energies were lower than − 5.0 kcal/mol, indicating that ligands and receptors could bind spontaneously.

**Fig. 7:**
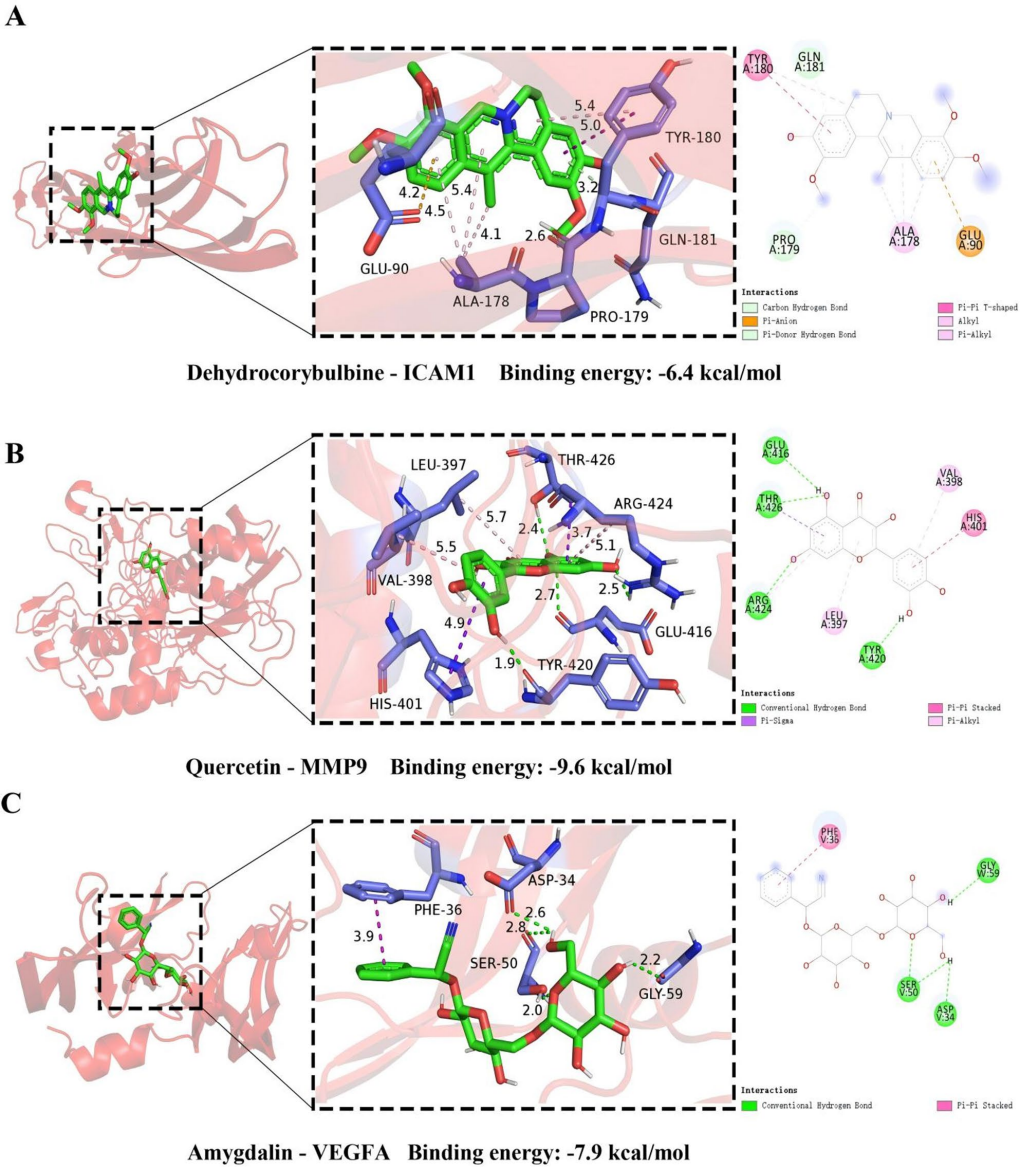
Schematic diagrams of docking between representative components and targets. **A** Dehydrocorybulbine and ICAM1 molecular docking visualization. **B** Quercetin and MMP9 molecular docking visualization. **C** Amygdalin and VEGFA visualization. Each protein-ligand interaction docking diagram is also divided into three small figures, figure on the left shows the overall binding position of ligand (sticks model) on related protein (cartoon model), the middle one represents the interaction of ligand (green) with important residues (purple) on related protein, the two-dimensional plan on the right shows the hydrogen bonds and hydrophobic interactions between ligand and protein residues. BE is short for binding energy

Then, for each target, representative component with strong binding affinity were selected for visualization. The results (Fig. 7) suggested that the dehydrocorybulbine could form one carbon hydrogen bond with the amino acid residue GLN181, one pi-donor hydrogen bond with PRO179, one pi-anion interaction with GLU90, one pi-pi t-shaped interaction with TYR180, four Alkyl/pi-Alkyl interactions with ALA178, TYR180 in ICAM1 protein. Quercetin could form one pi-sigma interaction with THR426, one pi-pi stacked interaction with HIS401, three pi-Alkyl interactions with VAL398, LEU397 and ARG424, as well as four conventional hydrogen bonds with GLU416, THR426, ARG424 and TYR420 in MMP9 protein. Amygdalin could form one pi-pi stacked interaction with PHE36, two conventional hydrogen bonds with SER50, ASP34 in chain V of VEGFA protein, besides, it could form one conventional hydrogen bond with GLY59 in chain W.

**Table 3:** The information of targets for molecular docking

Targets	PDB ID	Resolution	Structure	(X × Y × Z) /nm^3^	Center (X, Y, Z)
ICAM1	1IAM	2.1 Å	single stranded	35.25 × 26.25 × 30.0	53.0, 84.301, − 7.498
MMP9	1L6J	2.5 Å	single stranded	30.0 × 41.25 × 37.5	36.0, 38.0, 33.0
VEGFA	1BJ1	2.4 Å	double stranded	33.0 × 33.75 × 47.25	23.954, − 22.037, − 5.059

**Table 4:** Binding energy of molecular docking (kcal/mol)

Target	Component	Binding energy	Target	Component	Binding energy
ICAM1	Metformin (negative control)	−4.6	MMP9	Arachidonic Acid	−5.6
ICAM1	2-isopropyl-8-methylphenanthrene-3,4-dione	−6.3	MMP9	Neocryptotanshinone	−7.9
ICAM1	Cryptotanshinone	−6.2	MMP9	Caffeicacid	−7.9
ICAM1	6,7-dimethoxy-2-(2-phenylethyl) chromone	−6.2	MMP9	Luteolin	−7.8
ICAM1	Tanshinaldehyde	−6.0	MMP9	Danshenol B	−7.7
ICAM1	Dehydrocorybulbine	−6.4	MMP9	Pelargonidin	−7.7
MMP9	Metformin (negative control)	−5.8	MMP9	Isocorybulbine	−7.6
MMP9	5,6-dihydroxy-7-isopropyl-1,1-dimethyl-2,3-dihydrophenanthren-4-one	−9.0	MMP9	(2R)-3-(3,4-dihydroxyphenyl)-2-[(z)-3-(3,4-dihydroxyphenyl) acryloyl] oxy-propionic acid	−7.6
MMP9	Quercetin	−9.6	MMP9	Capaurine	−7.5
MMP9	2-(4-hydroxy-3-methoxyphenyl)-5-(3-hydroxypropyl)-7-methoxy-3-benzofurancarboxaldehyde	−7.2	MMP9	(Z)-3-[2-[(E)-2-(3,4-dihydroxyphenyl)vinyl]-3,4-dihydroxy-phenyl]acrylic acid	−7.4
MMP9	Salvianolic Acid G	−8.6	MMP9	Galeopsin	−7.3
MMP9	(S)-Scoulerine	−8.3	MMP9	Chaksine	−8.8
MMP9	Tanshinaldehyde	−8.1	MMP9	Sclareol	−7.2
MMP9	Isorhamnetin	−8.0	MMP9	Gamma-Decanolactone	−6.7
MMP9	Izoteolin	−8.0	VEGFA	Metformin (negative control)	−4.3
MMP9	Kaempferol	−7.9	VEGFA	Amygdalin	−7.9
MMP9	Myricanone	−7.9	VEGFA	(2R)-5,7-dihydroxy-2-(4-hydroxyphenyl) chroman-4-one	−7.2
MMP9	Salvianolic Acid J	−7.9			

#### MD simulation results

**Fig. 8:**
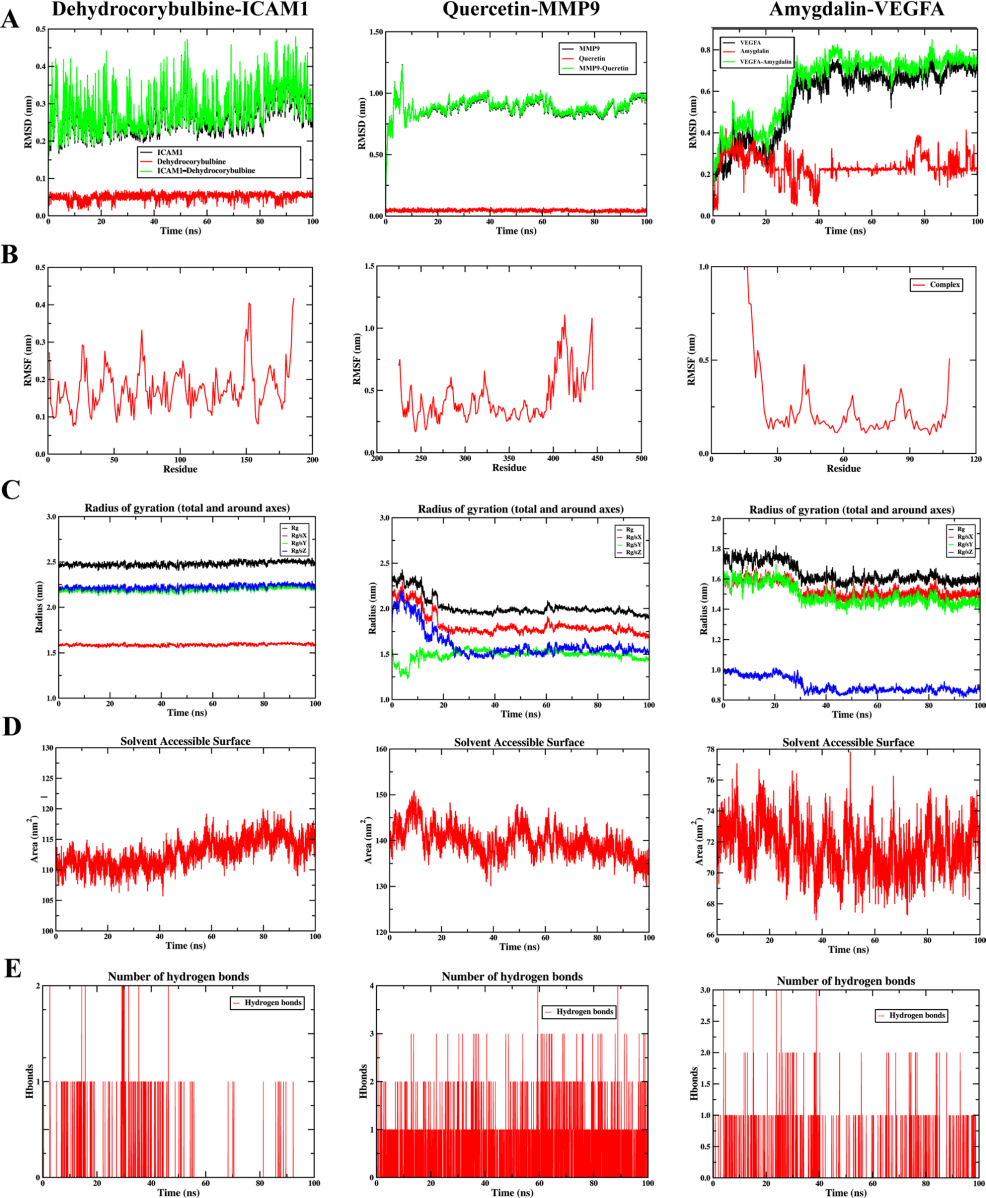
MD simulation results. Analysis of RMSD (**A**), RMSF (**B**), Rg (**C**), SASA (**D**), and hydrogen bonds (**E**) for ICAM1-dehydrocorybulbine, MMP9-quercetin and VEGFA-amygdalin complexes

RMSD is a reliable metric for assessing the conformational stability of protein and ligand. The final fluctuations of the RMSD curves for the three complex systems were within 0.1 nm, indicating that the systems had reached equilibrium (Fig. 8A). RMSF is an indicator for evaluating the dynamics of proteins. Results revealed that the RMSF values were relatively low in the binding regions and higher in the non-binding regions (Fig. 8B), suggesting that ligands influenced the flexibility and stability of the proteins. The Rg, a key parameter for evaluating the overall structural compactness, showed curves that eventually plateaued for all three complexes (Fig. 8C). These indicated that the systems attained dynamic equilibrium, with compact and stable overall conformations. SASA, which reflects the protein surface area, slightly decreased, implying that proteins were largely unaffected (Fig. 8D). Hydrogen-bond analysis showed that each ligand forms one to two persistent hydrogen bonds with the receptor in most of the trajectories, which further stabilized the complex (Fig. 8E).

### Animal model of EMs

**Fig. 9:**
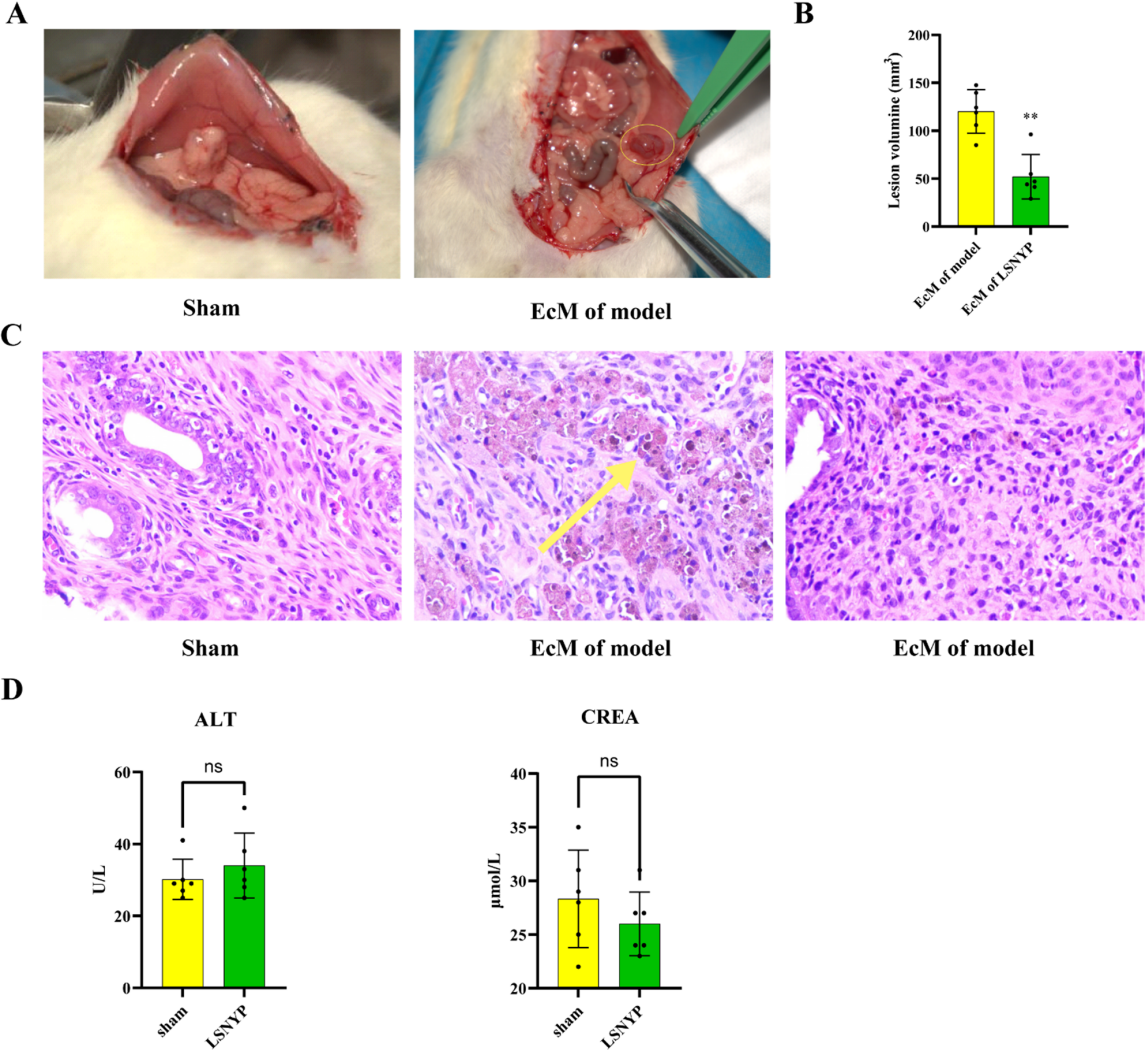
Animal model of EMs. **A** Modeling results. **B** Lesion volume; *n* = 6 (biological replicates), ^**^p < 0.01 vs. EuM of model group. **C** Effect of LSNYP on histopathology of lesions in rats (200×). **D** Effect of LSNYP on ALT and CREA. Note: Yellow arrows indicate macrophages containing hemosiderin

Uterine fragments were successfully implanted in the abdominal wall of rats, where these tissues formed cystic structures containing light yellow fluid with some surface and adjacent blood vessels (Fig. 9A). However, the volume of the lesion was significantly reduced after LSNYP treatment (Fig. 9B). Microscopic evaluation revealed that compared with the sham group, the EcM of model group contained irregularly arranged cells, some of which contained tawny cytoplasmic granules of hemosiderin; and the EcM of LSNYP exhibited more well-organized stromal cells and fewer inflammatory cells after treatment (Fig. 9C). In addition, the results of ALT and CREA suggested that there were no significant changes after LSNYP treatment (Fig. 9D).

### Effect of LSNYP on ICAM1, MMP9 and VEGFA expression was determined by immunohistochemistry

**Fig. 10:**
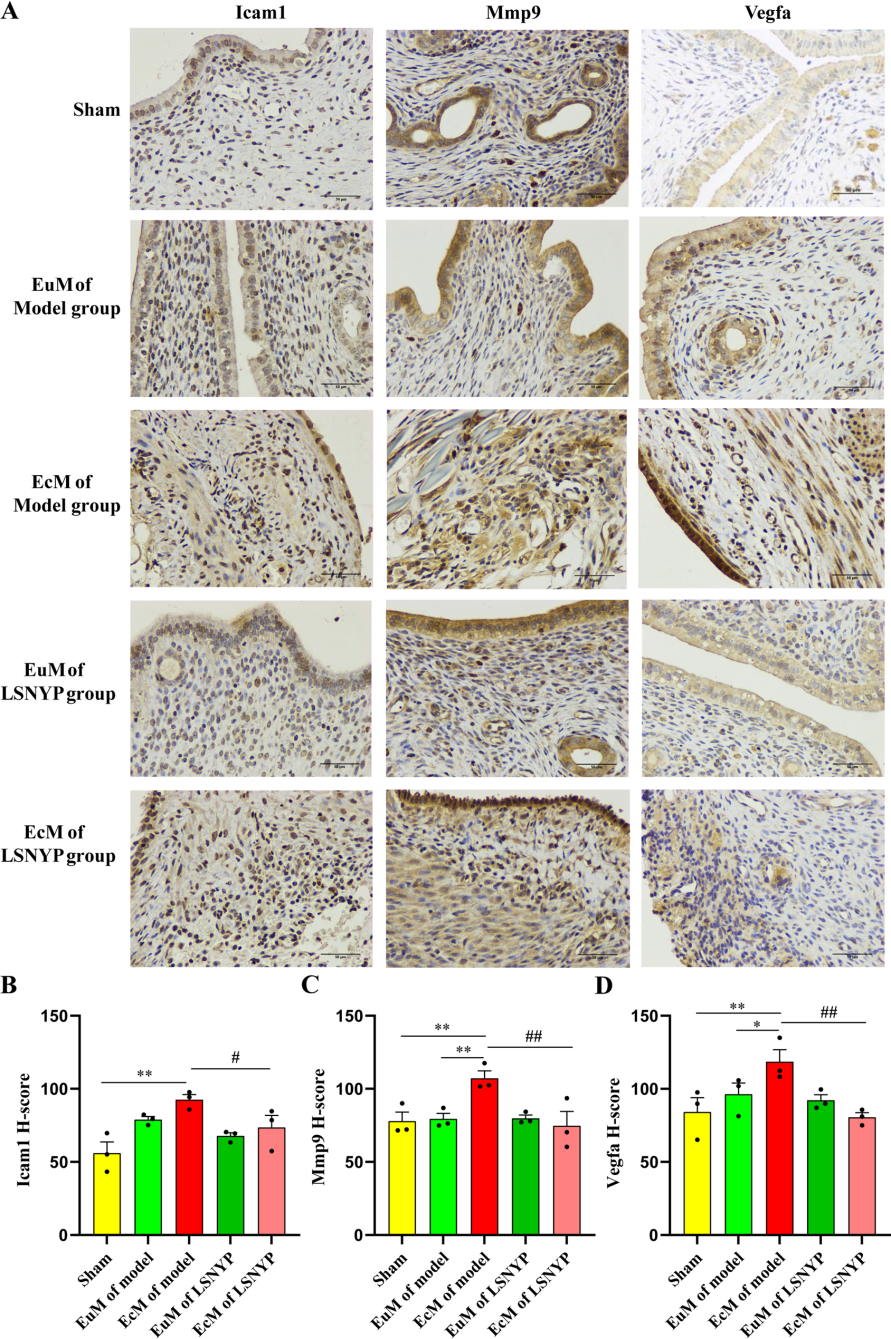
Effect of LSNYP on ICAM1, MMP9 and VEGFA was determined by immunohistochemistry (400×). **A** The representative stain images. The protein expression levels of ICAM1 (**B**), MMP9 (**C**) and VEGFA (**D**) in endometrial tissues. *n* = 3 (biological replicates). ^*^p < 0.05, and ^**^p < 0.01 vs. sham/EuM of model group; ^#^*p* < 0.05, and ^##^*p* < 0.01 vs. EcM of the model group

Compared with those found in the sham group (Fig. 10), the EuM of the model group showed increased expression levels of ICAM1, MMP9 and VEGFA, but the difference was not statistically significant. Compared with the levels in the sham group or the EuM of the model group, the EcM of the model group showed significantly upregulated expression of ICAM1, MMP9 and VEGFA (*P* < 0.05 or *P* < 0.01). However, LSNYP treatment significantly reduced ICAM1, MMP9 and VEGFA expression in the EcM of the model group (*P* < 0.05 or *P* < 0.01).

## Effect of LSNYP on ICAM1, MMP9 and VEGFA expression was determined by Western blotting

**Fig. 11:**
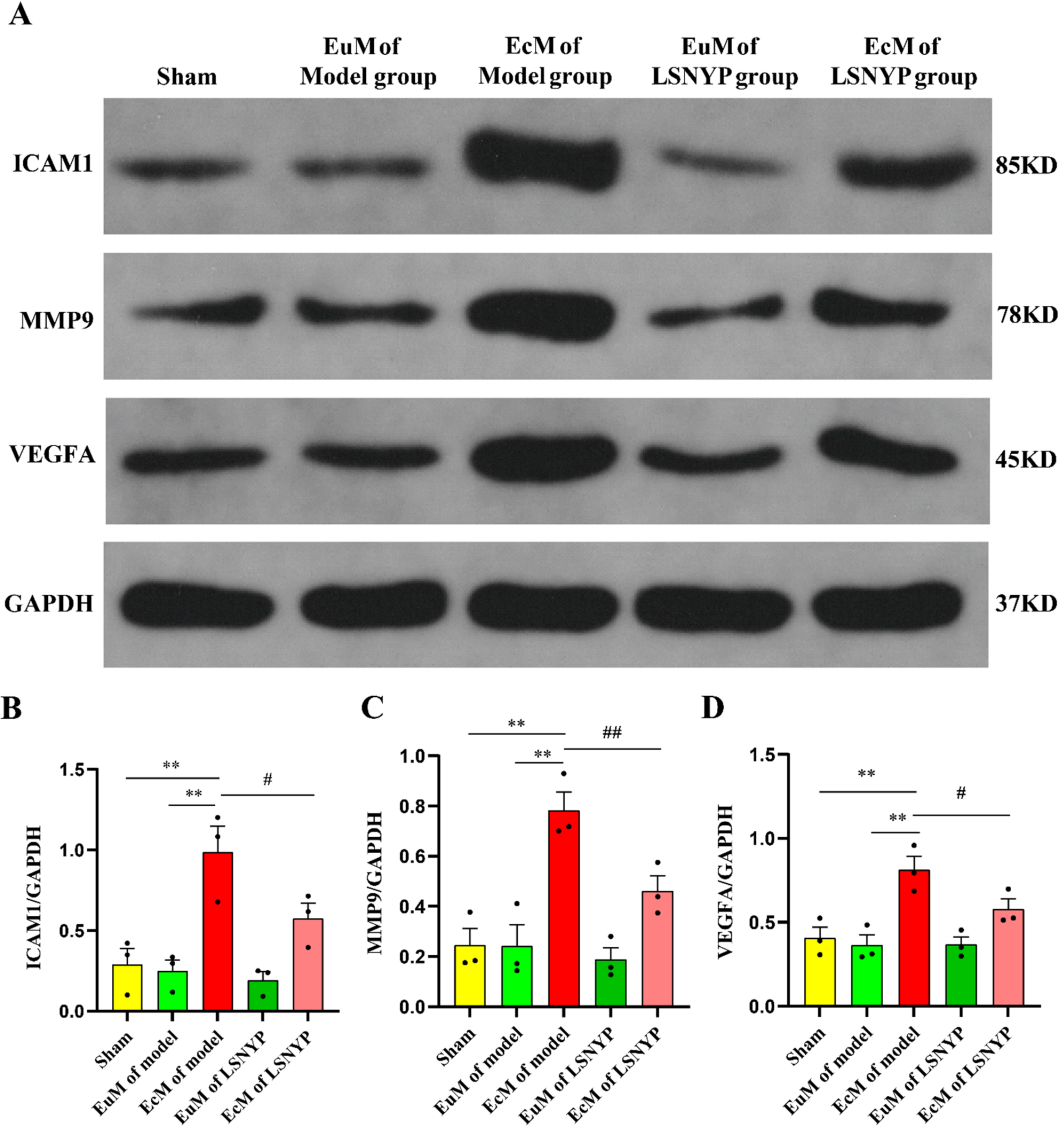
Effect of LSNYP on ICAM1, MMP9 and VEGFA was determined by western blotting. **A** The representative band. The protein expression levels of ICAM1 (**B**), MMP9 (**C**) and VEGFA (**D**) in endometrial tissues. *n* = 3 (biological replicates). ^**^p < 0.01 vs. sham/EuM of model group; ^#^*p* < 0.05, and ^##^*p* < 0.01 vs. EcM of the model group

Our studies (Fig. 11) demonstrated that ICAM1, MMP9 and VEGFA expression was significantly higher in the EcM of the model group than in the sham group and the EuM of the model group (*P* < 0.01); these results were consistent with the immunohistochemistry results. Moreover, the expression levels of ICAM1, MMP9 and VEGFA in the EcM of the LSNYP group were significantly reduced compared with those in the EcM of the model group (*P* < 0.05 or *P* < 0.01).

**Effect of LSNYP on** ***ICAM1***, **MMP9 and** ***VEGFA*** **gene expression**.

**Fig. 12:**
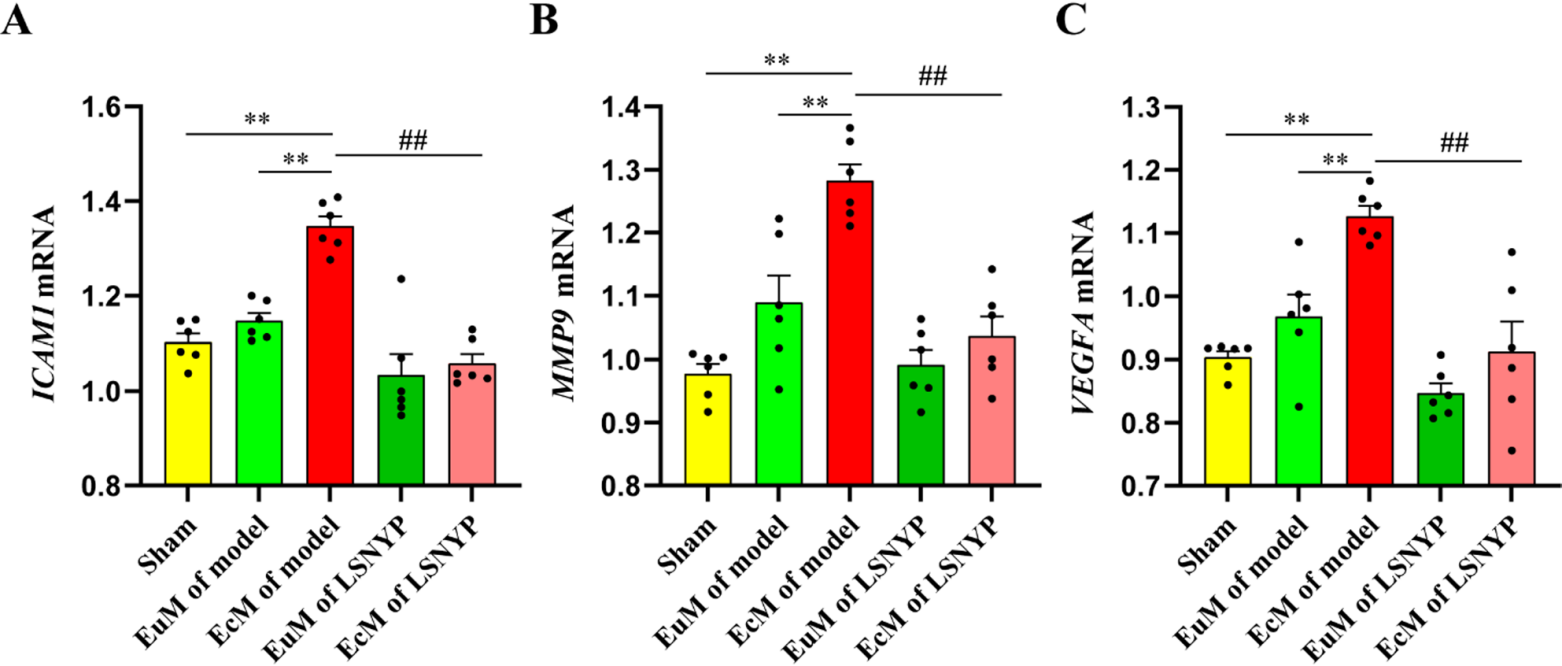
The mRNA expression of *ICAM1* (**A**), *MMP9* (**B**) and *VEGFA* (**C**) in endometrial tissues. *n* = 6 (biological replicates). ^**^p < 0.01 vs. sham/EuM of model group; ^##^*p* < 0.01 vs. EcM of the model group

The mRNA levels (Fig. 12) of *ICAM1*, *MMP9* and *VEGFA* were significantly higher in the EcM of the model group than in the sham group and the EuM of the model group (*P* < 0.01). In addition, the gene expression levels of *ICAM1*, *MMP9* and *VEGFA* in the EcM of the LSNYP group were significantly decreased compared with those in the EcM of the model group (*P* < 0.01).

### Effect of LSNYP on ESCs behavior

**Fig. 13:**
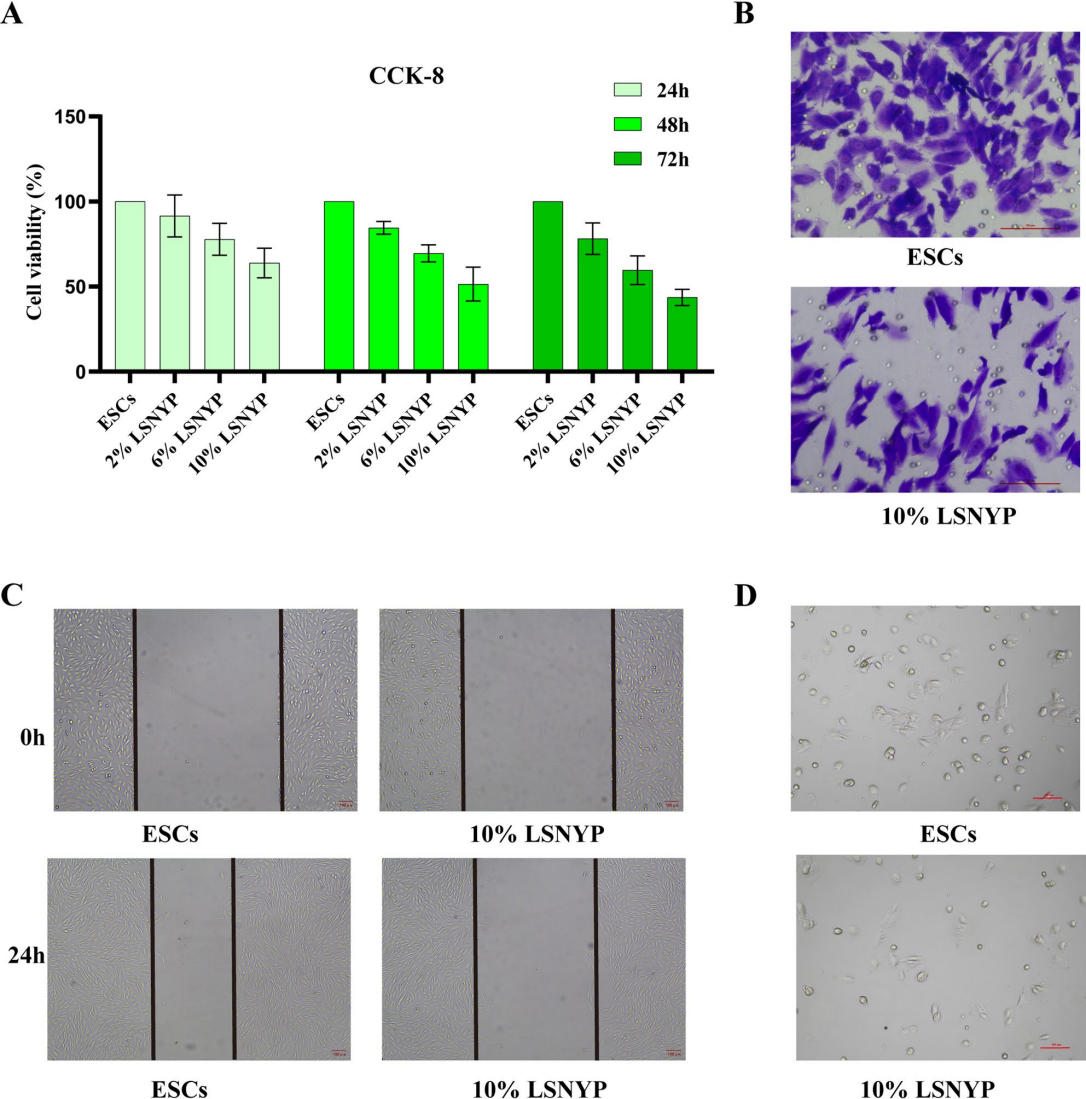
Effect of LSNYP on ESCs behavior. **A** Effect of different intervention times on cell viability. **B** Cell invasive assay (200×). **C** Cell scratch assay (40×). **D** Cell adhesion assay (100×)

CCK-8 analysis indicated that treatment with 10% LSNYP-containing serum for 72 h could more effectively inhibit the viability of ESCs (Fig. 13A), and this condition was therefore selected for subsequent experiments. After 72 h of 10% LSNYP-containing serum treatment, subsequent invasive assay revealed a reduction in the number of invasive cells in the 10% LSNYP group compared to the control ESCs group (Fig. 13B). Similarly, the wound healing rate was lower following LSNYP treatment (Fig. 13C). Furthermore, cell adhesion assay showed that exposure to 10% LSNYP-containing serum impaired the adhesive capacity of ESCs (Fig. 13D).

### Effect of LSNYP on core target genes in ESCs

**Fig. 14:**
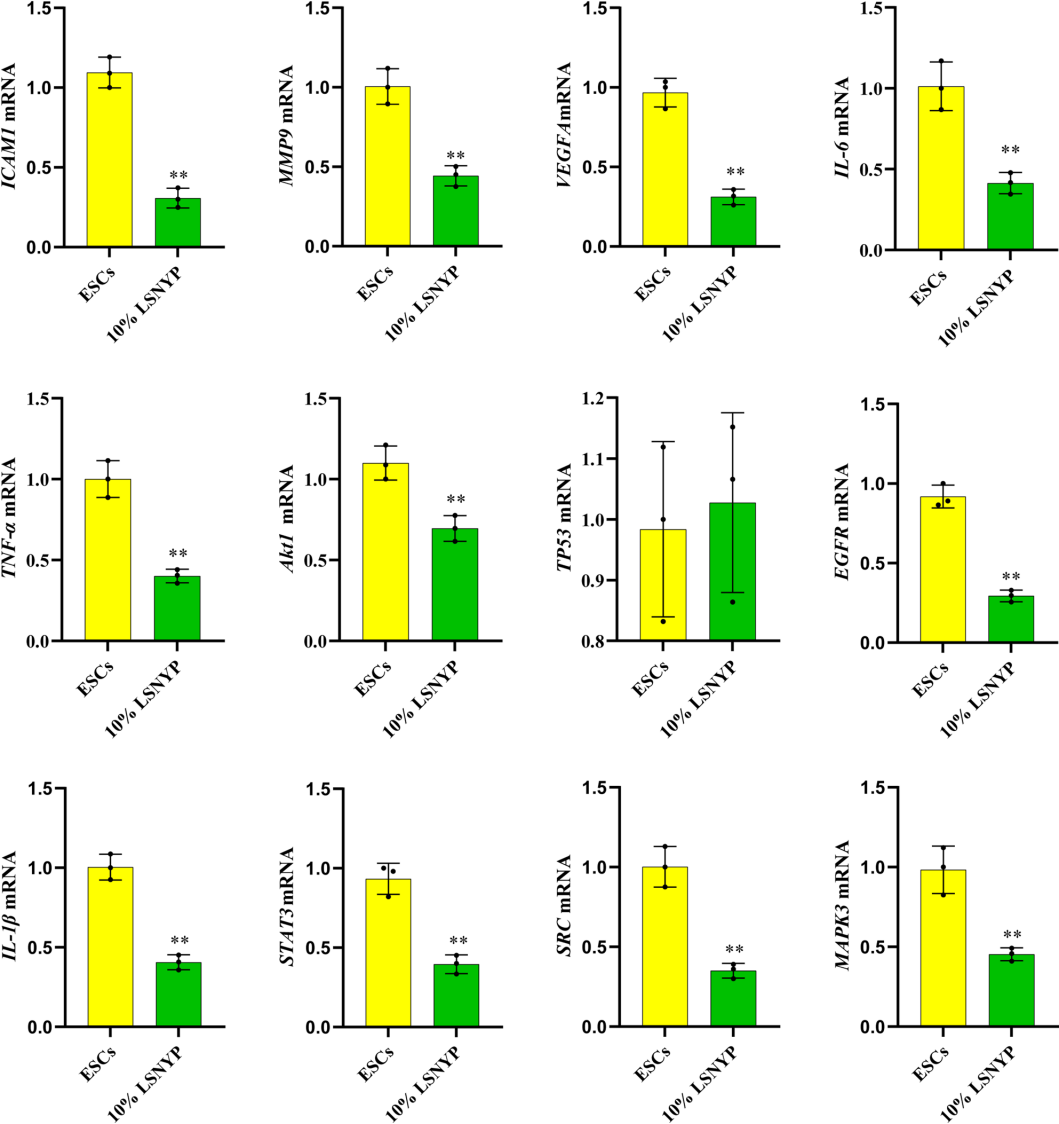
Effect of LSNYP on core target genes in ESCs. *n* = 3 (biological replicates). ^**^p < 0.01 vs. EScs group

The top 10 PPI network genes, and two additional core targets (*ICAM1*, *MMP9*) were validated by qPCR. Results (Fig. 14) indicated that 10% LSNYP-containing serum significantly reduced the mRNA levels of *ICAM1*, *MMP9*, *VEGFA*, *IL-6*, *TNF-α*, *Akt1*, *TP53*, *EGFR*, *IL-1β*, *STAT3*, *SRC*, and *MAPK3* in ESCs, whereas no significant effect was observed on *TP53.*

## Discussion

LSNYP, a popular TCM formula from Luo’s School of Gynecology, the composition follows the TCM theory of “Emperor-Minister-Assistant-Messenger”. The emperor herbs YMC and ZBM, which activate blood and dissipate masses, are responsible for treating the main symptoms. The assistant herbs TR, DS, CX, TBC and SZ remove blood stasis and promote blood regeneration; these herbs assist the emperor herbs by enhancing their effects. The assistant herbs PH, WLZ, WY and YHS provide further assistance by contributing to the promotion of chi and the relief of pain. WM, a messenger herb, expels corrupt matter and strongly counteracts the excessive promoting effects of other drugs. In this study, we utilized network pharmacology methods to build an herb-component-target network, facilitating an examination of its components and identification of crucial targets and pathways.

Our study identified 114 active components of LSNYP, including quercetin, kaempferol, luteolin, stigmasterol, ursolic acid, baicalin, amygdalin, and tanshinones. According to previous reports, quercetin inhibited PI3K/Akt, ERK1/2, and p38 MAPK pathways in endometriotic cells and exhibited antiproliferative and anti-inflammatory effects in mouse models [56]. Kaempferol inhibited migration and invasion of ishikawa cells, along with reduced expression of MMP9 [57]; it also suppressed tumorigenicity of endometrial cancer cells, inhibited tumor growth, and promoted apoptosis in a human endometrial cancer xenograft mouse model [58]. Luteolin inhibited the growth of 12Z human endometriotic cells and induced their apoptosis by activating caspases-3, -8, and − 9, and notably reduced expression of CCL2/CCL5 [59]. Stigmasterol not only induced human ovarian cancer cell death through activating the ER-mitochondrial axis [60], but also suppressed cell cycle progression, migration, and cancer stem cell activity in endometrial cancer cells by inhibiting the IGF1R/Akt/mTOR pathway [61]. Ursolic acid inhibited proliferation and capillary-like tube formation downregulated COX-2/PGE_2_ and VEGF, and activated JNK/p38 MAPK in ESCs [62]. Jun-Ya Ke et al. demonstrated that baicalein attenuated endometriotic invasiveness in vitro and in vivo by downregulating TGF-β1 secretion, thereby suppressing FURIN-MT1-MMP-mediated invasion of ESCs [63]. Amygdalin, the principal bioactive component of TR, could inhibit malignant activities of endometrial stromal cells and alleviates EMs via modulation of Wnt/β-catenin signaling [64]. Tanshinones have been shown to exert anticoagulant, anti-inflammatory, and antifibrotic properties [65]. Specifically, tanshinone IIA attenuated endometrial cell viability by upregulating caspase-3 and downregulating Bcl-2 in EMs [66, 67], while cryptotanshinone alleviated EMs-associated immunosuppression via JAK2/STAT3-mediated MDSC modulation [68]. Despite the potential for representativeness bias, the collective findings suggest that multiple medicinal components of LSNYP exert therapeutic effects on EMs by exerting anti-inflammatory activities, modulating immune and endocrine function, suppressing cell proliferation and angiogenesis, and other relevant pathways.

The KEGG pathway enrichment analysis revealed that LSNYP may regulate EMs through multiple signaling pathways, including those involved in the inflammatory response, endocrine system, angiogenesis, focal adhesion formation, and cell proliferation and apoptosis, etc. EMs lesions have characteristics of benign tumors; furthermore, TNF-α can promote EMs development [69, 70], and inhibition of the TNF signaling pathway can relieve the clinical symptoms of EMs [71]. EMs is related to dysfunction of endocrine axis, and excessive levels of estrogen and its related receptors can activate the MAPK and PI3K-Akt signaling pathways [72], and thus negatively affect EMs [73, 74]. The focal adhesion signaling pathway plays an important role in cell adhesion, migration and invasion [75]. The expression of focal adhesion kinase is positively correlated with the clinical stage of EMs and pelvic pain [76]. The AGE-RAGE signaling pathway in diabetic complications, fluid shear stress, atherosclerosis and EGFR tyrosine kinase inhibitor resistance are all involved in the expression of VEGF, which can promote angiogenesis and fibrosis in lesions [77]. GO enrichment results were similar to the KEGG, and indicating that LSNYP could regulate cell migration, adhesion, and proliferation as well as other biological processes.

Professor Jinghe Lang stated that the adhesion-invasion-angiogenesis progression represents a basic pathological change in the endometrium [52]. A previous study found that the levels of factors related to the progression could reflect the grade of EMs [78]. The generated PPI network showed that the core targets of LSNYP in EMs included Akt1, IL-6, VEGFA, TNF, ICAM1, MMP9, and PTGS2. Among these targets, ICAM1, MMP9 and VEGFA are core components of pathways (e.g., TNF and MAPK pathways), often engage in extensive protein interactions in PPI network, and are even directly and closely related to this adhesion-invasion-angiogenesis progression in EMs. ICAM1 belongs to the immunoglobulin superfamily of adhesion molecules. Studies have shown that patients with EMs have an abnormal pelvic environment, which can stimulate high expression of adhesion molecules such as ICAM1, leading to abnormal adhesion of cells to tissues [79]. MMP9, which belongs to the MMP family and can degrade type IV collagen [80], is the most representative invasive enzyme [81]. VEGFA is the most important angiogenic factor in the VEGF family and can promote the synthesis of interstitial collagenase and fibrinase in ectopic lesions [82].

To provide more evidence for the application of LSNYP, we verified the three core proteins through molecular docking and experimental studies. The results of molecular docking and MD simulation demonstrated that dehydrocorybulbine, quercetin or other active components stably bound to the ICAM1, MMP9 or VEGFA, suggesting some components of LSNYP may play a therapeutic role in EMs. Animal experiments showed LSNYP could shrink the lesion, improve the local pathological condition, and decrease the protein and gene expression levels of ICAM1, MMP9 and VEGFA in ectopic tissues of rats. In the modern pharmacological evaluation of TCM, drug-containing serum is widely used for in vitro experiments. Our results indicated that LSNYP-containing serum inhibited the viability of ESCs, reduced their invasive, migratory, and adhesive capacities, and downregulated genes associated with malignant behaviors, including *ICAM1*, *MMP9*, *VEGFA*, *IL-6*, *STAT3*, and *MAPK3*, as well as other relevant molecules. These findings suggest that LSNYP may exert therapeutic effects by inhibiting the adhesion-invasion-angiogenesis progression in EMs.

Taken together, our findings increase the knowledge of the mechanism underlying the effects of LSNYP in EMs, but there remain some limitations. First, our identification of components and targets from currently existing databases may bring a certain degree of representativeness bias and an incomplete list. Although ultrahigh-performance liquid chromatography-quadrupole-time-of-flight mass spectrometry (UHPLC-Q/TOF-MS) has been used in Guo’s thesis [43] (Supplement File 4), the quality markers of LSNYP-containing serum are still not very clear and require further research. Second, some differences between the EuM of model group and the sham group were not observed, which may be attributed to the limited impact of the transplanted lesions on the EuM, future modeling methods should be refined to better mimic the pathophysiological environment of EuM. Third, our findings on the three proteins and cell behaviors provide a foundation, the lack of pathway verification and potential off-target effects compromises the strength of the evidence. Subsequent studies should employ integrated transcriptomics and proteomics to accurately screen and verify the implicated pathways.

## Conclusions

In this study, network pharmacology analysis and experimental validation enabled a preliminary exploration of LSNYP’s therapeutic action against malignant biological behaviors in EMs. Our findings suggest LSNYP may be a promising candidate for EMs, potentially through inhibiting the adhesion-invasion-angiogenesis progression.

## Supplementary Information

Supplementary Material 1: 14 herbs of LSNYP.

Supplementary Material 2: 114 Potential active components.

Supplementary Material 3: 217 potential therapeutic targets.

Supplementary Material 4: Main quality control components were identified by UHPLC-Q/TOF-MS.

## Data Availability

All data generated or analyzed during this study are included in this article and the supplementary information files.

## Abbreviations

LSNYP: Luoshi neiyi prescription
EMs: Endometriosis
TCMSP: Traditional Chinese Medicine systems pharmacology database and analysis platform
BATMAN-TCM: Molecular mechanism of Traditional Chinese Medicine
GDA: Gene-disease association
PPI: Protein-protein interaction
GO: Gene ontology
KEGG: Kyoto encyclopedia of genes and genomes
ESCs: Endometriotic stromal cells
EcM: Ectopic endometrium
EuM: Eutopic endometrium
TCM: Traditional Chinese Medicine
OB: Oral bioavailability
DL: Drug-likeness
SMILES: Simplified molecular-input line-entry system
FDR: False discovery rate
MD: Molecular dynamics
TIP3P: Transferable intermolecular potential with 3 points
RMSD: Root-mean-square deviation
Rg: Radius of gyration
RMSF: Root-mean-square fluctuation
SASA: Solvent-accessible surface area
CREA: Creatinine
ALT: Aminotransferase
RT-PCR: Reverse transcription-polymerase chain reaction
H-score: Histochemistry score
SDS-PAGE: Sodium dodecyl sulfate-polyacrylamide gel electrophoresis
IPP: Image-pro plus
CCK-8: Cell counting kit-8
OD: Optical density
SD: Standard deviation
ANOVA: One-way analysis of variance
LSD: Least significant difference
BP: Biological process
MF: Molecular function
CC: Cellular component
UHPLC-Q/TOF-MS: Ultrahigh-performance liquid chromatography-quadrupole-time-of-flight mass spectrometry
