# Supplementary material for: Mechanisms of Luoshi Neiyi prescription (LSNYP) in endometriosis: a network pharmacology and experimental study
Source: Hereditas. 2026 Jan 19;163:24. doi: 10.1186/s41065-026-00637-2 (PMC12903665; doi:10.1186/s41065-026-00637-2)
Supplement: Supplementary file 1 — Supplementary Material 1: 14 herbs of LSNYP. [file 41065_2026_637_MOESM1_ESM.pdf]

Table S1: 14 herbs of a 250 mL of LSNYP

Chinese Name	Name for publishing	Amount (g)
Yimucao (YMC)	*Leonurus japonicus* Houtt. [Lamiaceae; *Herba Leonuri*]	33.5
Chuanxiong (CX)	*Conioselinum anthriscoides 'Chuanxiong'* [umbelliferae; *Chuanxiong*	10.25
Taoren (TR)	*Prunus persica* (L.) Batsch [Rosaceae; *Persicae Semen*]	17
Danshen (DS)	*Salvia miltiorrhiza* Bunge [Lamiaceae; *Salviae Miltiorrhizae Radix Et*	17
Puhuang (PH)	*Typha angustifolia* L. [Typhaceae; *Typhae Pollen*]	6.5
Yanhusuo (YHS)	*Corydalis yanhusuo* (Y.H.Chou & Chun C.Hsu) W.T.Wang ex Z.Y.Su & C.Y.Wu [Papaveraceae; *Corydalis Rhizoma*]	17
Wuyao (WY)	*Lindera aggregata* (Sims) Kosterm. [Lauraceae; *Linderae Radix*]	17
Haizao (HZ)	*Sargassum pallidum* (Turn.) C.Ag. [Sargassaceae; *Sargassum*]	33.5
Zhebeimu (ZBM)	*Fritillaria thunbergii* Miq. [Liliaceae; *Fritillariae Thunbergii Bulbus*]	17
Wumei (WM)	*Prunus mume* (Siebold) Siebold & Zucc. [Rosaceae; *Mume Fructus*]	11.25
Tubiechong (TBC)	*Eupolyphaga sinensis* Walker [Corydidae; *Eupolyphaga Steleophaga*]	13.5
Shanzha (SZ)	*Crataegus pinnatifida* Bunge [Rosaceae; *Crataegi Fructus*]	17
Wulingzhi (WLZ)	*Trogopterus xanthipes* Milne-Edwards [Talpidae; *Faeces Trogopterori*]	11.25
Muli (ML)	*Ostrea gigas* Thunberg [Ostreidae; *Ostreae Concha*]	28.25

Note: The preparation of LSNYP was carried out according to the following procedure: 14 herbal components (250 g) were combined and immersed in 2500 mL of water for 30 minutes. The mixture was first brought to a boil over high heat and then simmered gently for 1.5 hours. After filtration, the aqueous extract was collected. The residual herbal material was then refluxed with an additional 1250 mL of water for one hour under gentle boiling, followed by a second filtration. The two obtained extracts were pooled and condensed to a final volume of 250 mL. Subsequently, 0.125 g of ethylparaben and 0.75 g of sodium benzoate were added as preservatives. The finished product was finally bottled for storage at 4 °C until use.
