# Supplementary material for: Mechanisms of Luoshi Neiyi prescription (LSNYP) in endometriosis: a network pharmacology and experimental study
Source: Hereditas. 2026 Jan 19;163:24. doi: 10.1186/s41065-026-00637-2 (PMC12903665; doi:10.1186/s41065-026-00637-2)
Supplement: Supplementary file 2 — Supplementary Material 2: 114 Potential active components. [file 41065_2026_637_MOESM2_ESM.pdf]

Table S2: 114 Potential active components of LSNYP

No	Component	No	Component
1	Arachidonic Acid	58	Neocryptotanshinone Ii
2	Galeopsin	59	2-isopropyl-8-methylphenanthrene-3,4-dione
3	Zinc04073977	60	4-methylenemiltirone
4	Preleoheterin	61	Isoimperatorin
5	Quercetin	62	Sclareol
6	Isorhamnetin	63	Manool
7	Kaempferol	64	Isofucosterol
8	Pelargonidin	65	(2R)-5,7-dihydroxy-2-(4-hydroxyphenyl)chroman-4-one
9	Beta-Sitosterol	66	Wulingzhic Acid
10	Peimisine	67	Ruvoside
11	Ziebeimine	68	Caffeic acid dimethyl ether
12	Chaksine	69	Caffeicacid
13	Mandenol	70	12-oxoarundoin
14	Myricanone	71	1-ethyl-4,8-dimethoxy-beta-carboline
15	Senkyunone	72	Amygdalin
16	Wallichilide	73	Ursolicacid
17	Sitosterol	74	6,7-dimethoxy-2-(2-phenylethyl)chromone
18	Fa	75	Dmpec
19	α-Amyrin	76	Norboldine
20	Poriferasterol	77	C09495
21	Baicalin	78	Boldine
22	Poriferast-5-en-3beta-ol	79	Sitosterol Alpha1
23	Salvianolic Acid j	80	4a-formyl-7alpha-hydroxy-1-methyl-8-methylidene-4aalpha,4bbeta-gibbane-1alpha,10beta-dicarboxylic acid
24	Salvianolic Acid g	81	3-o-p-coumaroylquinic acid
25	Danshenol B	82	Hederagenin
26	Dan-Shexinkum d	83	Campesterol
27	Danshenol A	84	Stigmasterol
28	Tanshinaldehyde	85	Campest-5-en-3beta-ol
29	Przewalskin b	86	Coptisine
30	Dehydrotanshinone II A	87	Cryptopin
31	Tanshinone II A	88	Dihydrochelerythrine
32	Isotanshinone II	89	(S)-Scoulerine
33	2-(4-hydroxy-3-methoxyphenyl)-5-(3-hydroxypropyl)-7-methoxy-3-benzofurancarboxaldehyde	90	Cavidine
34	Cryptotanshinone	91	(R)-Canadine
35	Isocryptotanshi-None	92	Hyndarin
36	Salvilenone	93	Capaurine
37	Miltipolone	94	Clarkeanidine
38	1-methyl-8,9-dihydro-7h-naphtho[5,6-g]benzofuran-6,10,11-trione	95	Corydaline
39	Methylenetanshinquinone	96	Corydalmine
40	DihydrotanshinoneI	97	Corydine
41	1,2,5,6-tetrahydrotanshinone	98	Corynoloxine
42	(2R)-3-(3,4-dihydroxyphenyl)-2-[(Z)-3-(3,4-dihydroxyphenyl)acryloyl]oxy-propionic acid	99	Dehydrocavidine
43	Neocryptotanshinone	100	Dehydrocorybulbine
44	Miltionone I	101	Dehydrocorydaline

45	(E)-3-[2-(3,4-dihydroxyphenyl)-7-hydroxy-benzofuran-4-yl]acrylic acid	102	Izoteolin
46	Tanshinone VI	103	Isocorybulbine
47	5,6-dihydroxy-7-isopropyl-1,1-dimethyl-2,3-dihydrophenanthren-4-one	104	Leonticine
48	Deoxyneocryptotanshinone	105	N-methyllaurotetanine
49	Microstegiol	106	Pseudocoptisine
50	Sugiol	107	24240-05-9
51	(Z)-3-[2-[(E)-2-(3,4-dihydroxyphenyl)vinyl]-3,4-dihydroxy-phenyl]acrylic acid	108	Stylopine
52	Digallate	109	Tetrahydrocorysamine
53	Miltirone	110	St057701
54	C09092	111	Palmatine
55	Luteolin	112	Isocorypalmine
56	Salviolone	113	Bicuculline
57	Miltirone II	114	C09367
