# Supplementary material for: Mechanisms of Luoshi Neiyi prescription (LSNYP) in endometriosis: a network pharmacology and experimental study
Source: Hereditas. 2026 Jan 19;163:24. doi: 10.1186/s41065-026-00637-2 (PMC12903665; doi:10.1186/s41065-026-00637-2)
Supplement: Supplementary file 3 — Supplementary Material 3: 217 potential therapeutic targets. [file 41065_2026_637_MOESM3_ESM.pdf]

Table S3: 217 potential therapeutic targets of LSNYP in EMs

No	Protein	Symbol	No	Protein	Symbol
1	C-C chemokine receptor type 1	CCR1	110	Tyrosine-protein kinase TIE-2	TEK
2	Apoptosis regulator Bcl-X	BCL2L1	111	Thyroid hormone receptor alpha	THRA
3	Matrix metalloproteinase 3	MMP3	112	C-C chemokine receptor type 2	CCR2
4	Matrix metalloproteinase 9	MMP9	113	Gonadotropin-releasing hormone receptor	GNRHR
5	Matrix metalloproteinase 1	MMP1	114	Carnitine palmitoyltransferase 2	CPT2
6	Cyclooxygenase-2	PTGS2	115	Cathepsin (B and K)	CTSB
7	Prostaglandin E synthase	PTGES	116	Neuronal acetylcholine receptor protein alpha-7 subunit	CHRNA7
8	Complement factor D	CFD	117	c-Jun N-terminal kinase 2	MAPK9
9	Phospholipase A2 group IIA	PLA2G2A	118	Monoamine oxidase B	MAOB
10	Tyrosine-protein kinase receptor UFO	AXL	119	Sphingosine 1-phosphate receptor Edg-1	S1PR1
11	Oxytocin receptor	OXTR	120	Bradykinin B1 receptor	BDKRB1
12	Serine/threonine-protein kinase Aurora-A	AURKA	121	Apoptosis regulator Bcl-2	BCL2
13	Leucine-rich repeat serine/threonine-protein kinase 2	LRRK2	122	Histone deacetylase 1	HDAC1
14	Epidermal growth factor receptor erbB1	EGFR	123	TNF-alpha	TNF
15	Tyrosine-protein kinase SRC	SRC	124	Interleukin-2	IL2
16	Focal adhesion kinase 1	PTK2	125	Aldehyde dehydrogenase	ALDH2
17	Tyrosine-protein kinase JAK1	JAK1	126	Arylamine N-acetyltransferase 1	NAT1
18	Hepatocyte growth factor receptor	MET	127	Glutathione S-transferase Pi	GSTP1
19	Serine/threonine-protein kinase MST1	STK4	128	TGF-beta receptor type I	TGFBR1
20	Cathepsin D	CTSD	129	Dual specificity protein phosphatase 1	DUSP1
21	Cyclooxygenase-1	PTGS1	130	Intercellular adhesion molecule-1	ICAM1
22	Cytochrome P450 19A1	CYP19A1	131	Histone deacetylase 2	HDAC2
23	Steroid 5-alpha-reductase 1	SRD5A1	132	Fibroblast growth factor receptor 1	FGFR1
24	Steroid 5-alpha-reductase 2	SRD5A2	133	Steryl-sulfatase	STS
25	C-C chemokine receptor type 5	CCR5	134	Nerve growth factor receptor Trk-A	NTRK1
26	Glucocorticoid receptor	NR3C1	135	Isocitrate dehydrogenase [NADP] cytoplasmic	IDH1
27	Progesterone receptor	PGR	136	Bradykinin B2 receptor	BDKRB2
28	Cytochrome P450 17A1	CYP17A1	137	Serine/threonine-protein kinase mTOR	MTOR
29	Estrogen receptor beta	ESR2	138	Transforming protein p21/H-Ras-1	HRAS
30	Mineralocorticoid receptor	NR3C2	139	Thymidine phosphorylase	TYMP
31	Vanilloid receptor	TRPV1	140	Serine/threonine-protein kinase PAK 4	PAK4
32	Corticosteroid binding globulin	SERPINA6	141	Urokinase-type plasminogen activator	PLAU
33	Testis-specific androgen-binding protein	SHBG	142	C-C chemokine receptor type 9	CCR9
34	Dual specificity phosphatase Cdc25A	CDC25A	143	Insulin-like growth factor binding protein 3	IGFBP3
35	Estradiol 17-beta-dehydrogenase 2	HSD17B2	144	ELAV-like protein 1	ELAVL1

36	Cytochrome P450 2C19	CYP2C19	145	Methyl-CpG-binding domain protein 2	MBD2
37	Sulfonylurea receptor 2	ABCC9	146	Plasminogen activator inhibitor-1	SERPINE1
38	Monoamine oxidase A	MAOA	147	Estrogen-related receptor beta	ESRRB
39	Vitamin D receptor	VDR	148	G-protein coupled estrogen receptor 1	GPER1
40	MAP kinase ERK2	MAPK1	149	Hematopoietic cell protein-tyrosine phosphatase 70Z-PEP	PTPN22
41	Interleukin-6 receptor subunit beta	IL6ST	150	Vascular cell adhesion protein 1	VCAM1
42	Myeloperoxidase	MPO	151	Carbonyl reductase [NADPH] 1	CBR1
43	Heme oxygenase 1 (by homology)	HMOX1	152	Methionine aminopeptidase 2	METAP2
44	Indoleamine 2,3-dioxygenase	IDO1	153	LIM domain kinase 1	LIMK1
45	Xanthine dehydrogenase	XDH	154	DNA (cytosine-5)-methyltransferase 3A	DNMT3A
46	Insulin-like growth factor I receptor	IGF1R	155	RAS guanyl-releasing protein 1	RASGRP1
47	Thrombin	F2	156	Adenosine deaminase	ADA
48	Serine/threonine-protein kinase Aurora-B	AURKB	157	Vesicular acetylcholine transporter	SLC18A3
49	Death-associated protein kinase 1	DAPK1	158	Cytochrome P450 3A4	CYP3A4
50	Glycogen synthase kinase-3 beta	GSK3B	159	Coagulation factor VII/tissue factor	F3
51	Vascular endothelial growth factor receptor 2	KDR	160	Placenta growth factor	PGF
52	Matrix metalloproteinase 13	MMP13	161	Vascular endothelial growth factor A	VEGFA
53	Arachidonate 15-lipoxygenase	ALOX15	162	Cell division control protein 42 homolog	CDC42
54	Serine/threonine-protein kinase PLK1	PLK1	163	15-hydroxyprostaglandin dehydrogenase [NAD+]	HPGD
55	Matrix metalloproteinase 2	MMP2	164	NAD-dependent deacetylase sirtuin 1	SIRT1
56	Interleukin-8 receptor A	CXCR1	165	Ezrin	EZR
57	Serine/threonine-protein kinase AKT	AKT1	166	Caspase-9	CASP9
58	Cytochrome P450 1B1	CYP1B1	167	Transforming growth factor beta-1	TGFB1
59	ATP-binding cassette sub-family G member 2	ABCG2	168	Cytochrome P450 1A1	CYP1A1
60	DNA topoisomerase II alpha	TOP2A	169	Tyrosine 3-hydroxylase	TH
61	DNA-(apurinic or apyrimidinic site) lyase	APEX1	170	Inhibitor of NF-kappa-B kinase (IKK)	CHUK
62	Cyclin-dependent kinase 6	CDK6	171	Macrophage-stimulating protein receptor	MST1R
63	Estradiol 17-beta-dehydrogenase 1	HSD17B1	172	Nitric-oxide synthase, endothelial	NOS3
64	Aryl hydrocarbon receptor	AHR	173	Lipoxin A4 receptor	FPR2
65	Matrix metalloproteinase 12	MMP12	174	Baculoviral IAP repeat-containing protein 2	BIRC2
66	Telomerase reverse transcriptase	TERT	175	Tumor necrosis factor receptor R1	TNFRSF1A
67	adipocyte	FABP4	176	Fibroblast growth factor receptor 2	FGFR2
68	Peroxisome proliferator-activated receptor gamma	PPARG	177	EZH2/SUZ12/EED/RBBP7/RBBP4	EZH2
69	Cytochrome P450 26A1	CYP26A1	178	cGMP-dependent protein kinase 1 beta	PRKG1
70	Protein-tyrosine phosphatase 2C	PTPN11	179	(BAD)	BAD
71	Retinoic acid receptor beta	RARB	180	Phosphodiesterase 1C	PDE1C

72	Leukotriene B4 receptor 1	LTB4R	181	Tumour suppressor p53/oncoprotein Mdm2	TP53
73	Plasma retinol-binding protein	RBP4	182	Dual specificity mitogen-activated protein kinase kinase 7	MAP2K7
74	Estrogen receptor alpha	ESR1	183	Acidic fibroblast growth factor	FGF1
75	Nuclear receptor ROR-gamma	RORC	184	Heparanase	HPSE
76	Cathepsin G	CTSG	185	Basic fibroblast growth factor	FGF2
77	Prostanoid EP3 receptor	PTGER3	186	Galectin-3	LGALS3
78	Plasminogen	PLG	187	Galectin-9	LGALS9
79	LXR-beta	NR1H2	188	Macrophage colony stimulating factor receptor	CSF1R
80	Low molecular weight phosphotyrosine protein phosphatase	ACP1	189	HMG-CoA reductase	HMGCR
81	Hypoxia-inducible factor 1 alpha	HIF1A	190	Androgen Receptor	AR
82	Protein kinase C beta	PRKCB	191	Tyrosine-protein kinase TYK2	TYK2
83	Cytochrome P450 24A1	CYP24A1	192	Mu opioid receptor	OPRM1
84	Interleukin-1 beta	IL1B	193	MAP kinase p38 alpha	MAPK14
85	Nuclear receptor subfamily 4 group A member 1	NR4A1	194	Aldo-keto-reductase family 1 member C3	AKR1C3
86	Matrix metalloproteinase 7	MMP7	195	Cyclin-dependent kinase 1	CDK1
87	Caspase-1	CASP1	196	MAP kinase ERK1	MAPK3
88	Matrix metalloproteinase 14	MMP14	197	Arachidonate 5-lipoxygenase	ALOX5
89	Macrophage migration inhibitory factor	MIF	198	Aldose reductase	AKR1B1
90	Glutathione S-transferase Mu 1	GSTM1	199	Cannabinoid receptor 1	CNR1
91	Proto-oncogene c-JUN	JUN	200	Prostanoid EP2 receptor	PTGER2
92	Rho-associated protein kinase 2	ROCK2	201	Prostanoid EP4 receptor	PTGER4
93	Selectin E	SELE	202	Nitric oxide synthase, inducible	NOS2
94	Leukocyte elastase	ELANE	203	Angiotensin-converting enzyme	ACE
95	DNA (cytosine-5)-methyltransferase 3B	DNMT3B	204	Serotonin transporter	SLC6A4
96	Caspase-8	CASP8	205	Dopamine D2 receptor	DRD2
97	Interleukin-6	IL6	206	Neprilysin	MME
98	Cholesteryl ester transfer protein	CETP	207	Vascular endothelial growth factor receptor 1	FLT1
99	Rho-associated protein kinase 1	ROCK1	208	Type-1 angiotensin II receptor	AGTR1
100	Receptor protein-tyrosine kinase erbB-2	ERBB2	209	Aldo-keto reductase family 1 member C2	AKR1C2
101	Ephrin type-A receptor 3	EPHA3	210	Aldo-keto reductase family 1 member C1	AKR1C1
102	Signal transducer and activator of transcription 3	STAT3	211	Neurokinin 1 receptor	TACR1
103	Interleukin-8 receptor B	CXCR2	212	Epoxide hydrolase 1	EPHX1
104	Signal transducer and activator of transcription 6	STAT6	213	Corticotropin releasing factor receptor 1	CRHR1
105	Serine/threonine-protein kinase RAF	RAF1	214	PI3-kinase p110-alpha/p85-alpha	PIK3CA
106	Serine/threonine-protein kinase B-raf	BRAF	215	P2X purinoceptor 3	P2RX3
107	ADAM17	ADAM17	216	Catechol O-methyltransferase	COMT
108	Ribosomal protein S6 kinase 1	RPS6KB1	217	Endothelin receptor ET-A	EDNRA
109	C-X-C chemokine receptor type 3	CXCR3			
