# Supplementary material for: Mechanisms of Luoshi Neiyi prescription (LSNYP) in endometriosis: a network pharmacology and experimental study
Source: Hereditas. 2026 Jan 19;163:24. doi: 10.1186/s41065-026-00637-2 (PMC12903665; doi:10.1186/s41065-026-00637-2)
Supplement: Supplementary file 4 — Supplementary Material 4: Main quality control components were identified by UHPLC-Q/TOF-MS. [file 41065_2026_637_MOESM4_ESM.pdf]

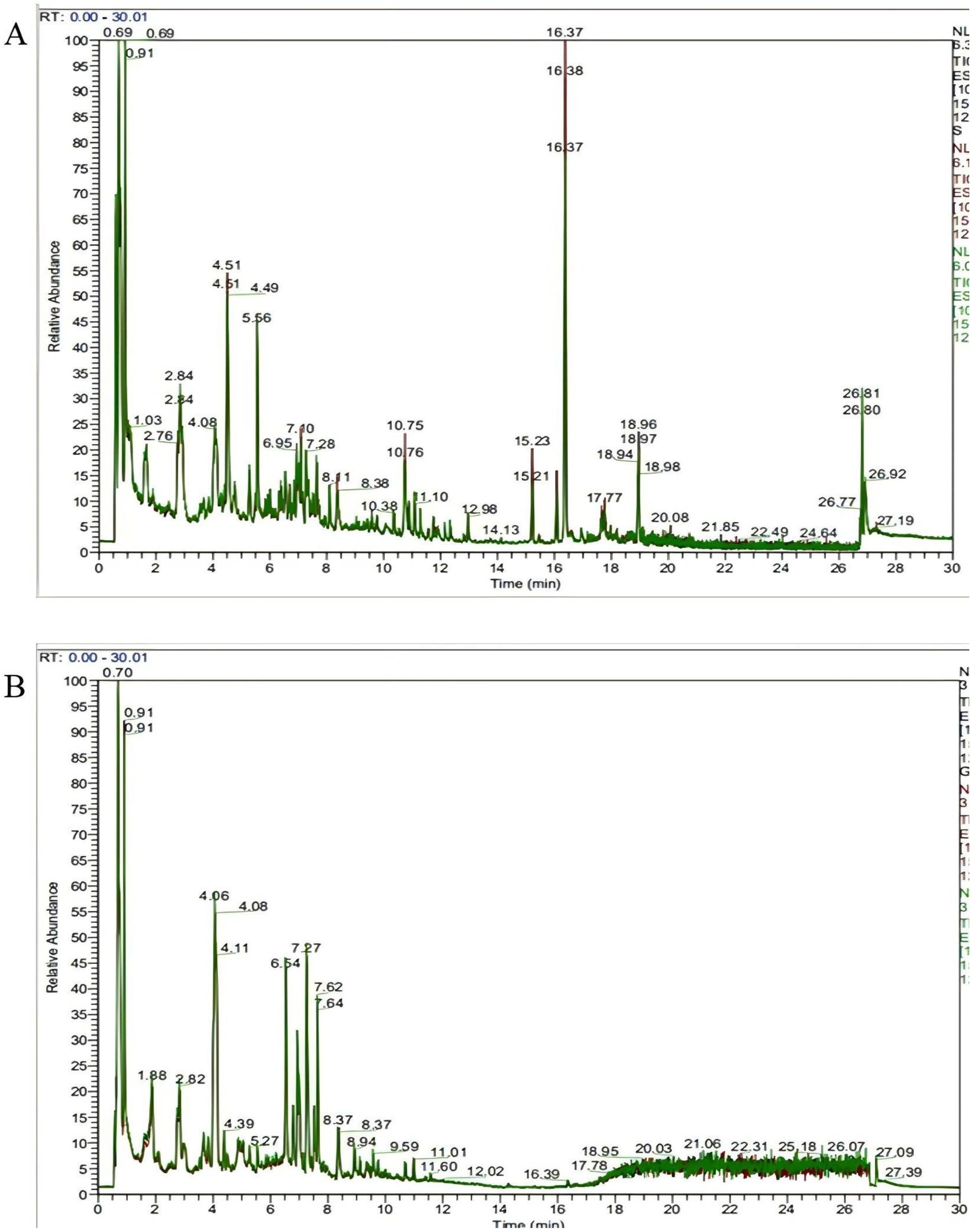

Figure S1: UHPLC-ESI-Q-MS chromatograms of LSNYP obtained in positive (A) and negative (B) ion modes.

Table S4: main quality control components were identified in LSNYP

NO	Retention time (min)	Identification	Formula
1	0.51	Paeonol	C9H10O3
2	0.66	Mannitol	C6H14O6
3	0.76	Stachydrine	C7H13NO2
4	1.57	Tetramethylpyrazine	C8H12N2
5	1.83	5-Hydroxymethylfurfural	C6H6O3
6	1.84	Danshensu	C9H10O5
7	2.08	Protocatechuic acid	C7H6O4
8	3.18	Chlorogenic acid	C16H18O9
9	4.21	Higenamine	C16H17NO3
10	5.03	Malic acid	C4H6O5
11	5.26	Amygdalin	C20H27NO11
12	5.57	Leonurine	C14H21N3O5
13	5.86	Typhaneoside	C34H42O20
14	5.91	Hyperoside	C21H20O12
15	6.22	Lithospermic acid	C27H22O12
16	6.27	Isorhamnetin-3-Onehesperidine	C28H32O16
17	7.00	Salvianolic acid A	C26H22O10
18	7.36	Edpetiline	C33H53NO8
19	7.69	Isoferulic acid	C10H10O4
20	7.72	resveratrol	C14H12O3
21	7.81	Peimine	C27H45NO3
22	9.98	Curcumin	C21H20O6
23	11.19	Senkyunolide A	C12H16O2
